# Genetic analysis for a shared biological basis between migraine and coronary artery disease

**DOI:** 10.1212/NXG.0000000000000010

**Published:** 2015-07-02

**Authors:** Bendik S. Winsvold, Christopher P. Nelson, Rainer Malik, Padhraig Gormley, Verneri Anttila, Jason Vander Heiden, Katherine S. Elliott, Line M. Jacobsen, Priit Palta, Najaf Amin, Boukje de Vries, Eija Hämäläinen, Tobias Freilinger, M. Arfan Ikram, Thorsten Kessler, Markku Koiranen, Lannie Ligthart, George McMahon, Linda M. Pedersen, Christina Willenborg, Hong-Hee Won, Jes Olesen, Ville Artto, Themistocles L. Assimes, Stefan Blankenberg, Dorret I. Boomsma, Lynn Cherkas, George Davey Smith, Stephen E. Epstein, Jeanette Erdmann, Michel D. Ferrari, Hartmut Göbel, Alistair S. Hall, Marjo-Riitta Jarvelin, Mikko Kallela, Jaakko Kaprio, Sekar Kathiresan, Terho Lehtimäki, Ruth McPherson, Winfried März, Dale R. Nyholt, Christopher J. O'Donnell, Lydia Quaye, Daniel J. Rader, Olli Raitakari, Robert Roberts, Heribert Schunkert, Markus Schürks, Alexandre F.R. Stewart, Gisela M. Terwindt, Unnur Thorsteinsdottir, Arn M.J.M. van den Maagdenberg, Cornelia van Duijn, Maija Wessman, Tobias Kurth, Christian Kubisch, Martin Dichgans, Daniel I. Chasman, Chris Cotsapas, John-Anker Zwart, Nilesh J. Samani, Aarno Palotie, For the CARDIoGRAM Consortium and the International Headache Genetics Consortium

**Affiliations:** Author affiliations are provided at the end of the article.

## Abstract

**Objective:**

To apply genetic analysis of genome-wide association data to study the extent and nature of a shared biological basis between migraine and coronary artery disease (CAD).

**Methods:**

Four separate methods for cross-phenotype genetic analysis were applied on data from 2 large-scale genome-wide association studies of migraine (19,981 cases, 56,667 controls) and CAD (21,076 cases, 63,014 controls). The first 2 methods quantified the extent of overlapping risk variants and assessed the load of CAD risk loci in migraineurs. Genomic regions of shared risk were then identified by analysis of covariance patterns between the 2 phenotypes and by querying known genome-wide significant loci.

**Results:**

We found a significant overlap of genetic risk loci for migraine and CAD. When stratified by migraine subtype, this was limited to migraine without aura, and the overlap was protective in that patients with migraine had a lower load of CAD risk alleles than controls. Genes indicated by 16 shared risk loci point to mechanisms with potential roles in migraine pathogenesis and CAD, including endothelial dysfunction (*PHACTR1*) and insulin homeostasis (*GIP*).

**Conclusions:**

The results suggest that shared biological processes contribute to risk of migraine and CAD, but surprisingly this commonality is restricted to migraine without aura and the impact is in opposite directions. Understanding the mechanisms underlying these processes and their opposite relationship to migraine and CAD may improve our understanding of both disorders.

Migraine affects 19% of women and 11% of men worldwide and causes more years lost to disability than any other neurologic disorder.^1,2^ In about one-third of patients, headache attacks are preceded by transient neurologic symptoms termed migraine aura, and migraine with and without aura (MA and MO, respectively) are believed to have a partially distinct pathogenic basis.^3^ It has long been assumed that the vascular system is involved in migraine pathogenesis, but little is known of the specific biological processes involved, and the relative importance of neuronal and vascular mechanisms remains controversial.^3–6^ Supporting a vascular basis, epidemiologic studies have found an increased risk for stroke among patients with migraine, most pronounced for MA.^7^ Some recent studies indicate a similar risk increase for coronary artery disease (CAD), the most common vascular disorder, although the association is less certain than for stroke.^8–11^ This raises the question of whether migraine and cardiovascular disease have a shared biological basis.

Both migraine and CAD have a strong genetic determination, and recent genome-wide association studies (GWAS) have identified risk variants for each. If migraine and CAD have a shared biological basis, one might anticipate that they will also share genetic variants that affect their risk. In this study, we utilized data from 2 large-scale nonoverlapping GWAS meta-analyses of migraine (the International Headache Genetics Consortium, IHGC)^12^ and CAD (Coronary ARtery DIsease Genome-Wide Replication And Meta-Analysis, CARDIoGRAM)^13^ to quantify shared genetic risk.

## METHODS

### Study cohorts.

**Figure 1:**
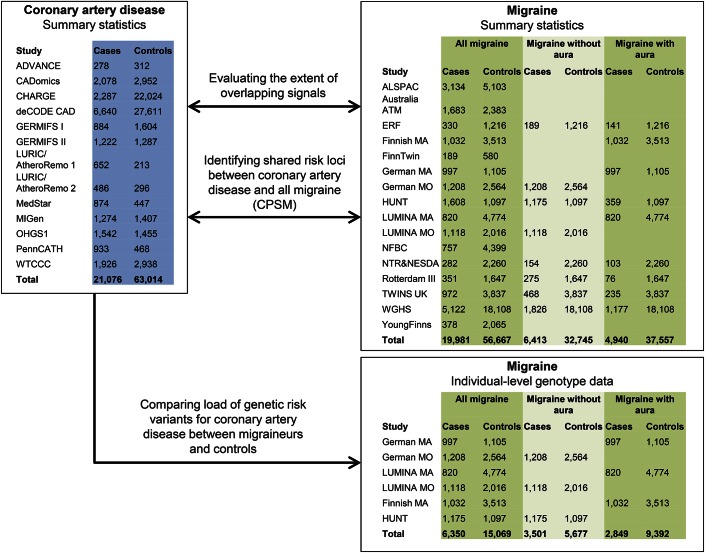
Study design and included cohorts CPSM = Cross-Phenotype Spatial Mapping.

Summary statistics (*p* value and effect size) at single nucleotide polymorphism (SNP) level from 2 recently performed meta-analyses of genome-wide association data on migraine (IHGC)^12^ and CAD (CARDIoGRAM)^13^ were used in the present study. After excluding overlapping samples, the 2 studies consisted of 19,981 cases with migraine vs 56,667 controls, and 21,076 cases with CAD vs 63,014 controls. A proportion of the migraine cases were phenotyped in sufficient detail to allow subclassification into MO (6,413 cases, 32,745 controls) and MA (4,940 cases, 37,557 controls). In addition, individual-level genotype data were available for a proportion of the migraine cohorts (6,350 migraine cases vs 15,069 controls from the German MA and MO cohorts, Dutch LUMINA study, Finnish MA study, and the HUNT Study, Norway). All data sets were imputed by using the HapMap release 21 or 22 as reference. An overview of the study design and the included cohorts is given in figure 1. A detailed description of samples, genotyping, and association analyses is given in e-Methods, tables e-1 and e-2, and figure e-1 at Neurology.org/ng.

### Standard protocol approvals, registrations, and patient consents.

For all study cohorts, participation was based on informed consent. Each study was approved by local research ethics boards in the country where the study cohort was collected. See original publications of the 2 studies for full details of ethics and consent procedures.^12,13^

### Analytic approach.

#### Evaluating extent of overlapping signals.

To assess whether more association signals were shared between the migraine and CAD studies than would be expected by chance, we used a set of 2,342,101 overlapping SNPs that were directly typed or imputed in both studies. Following the same procedure as described in a previous study,^14^ we first sorted the SNPs by association *p* value to migraine. Starting from the top of the list, all subsequent SNPs with linkage disequilibrium (LD) *r*^2^ > 0.05 (based on HapMap CEU release 27) were removed. This process was repeated until a set of 92,654 SNPs in approximate linkage equilibrium remained. For each of 5 separate *p* value cutoffs (1 × 10^−2^, 1 × 10^−3^, 1 × 10^−4^, 1 × 10^−5^, and 1 × 10^−6^), we counted the number of SNPs above and below the cutoff in each of the 2 studies, resulting in one 2 × 2 table for each *p* value cutoff. The Fisher exact test was used to estimate deviation from the expected distribution, and false discovery rate correction was performed on all 6 tests using the p.adjust function in *R*.^15^ A corrected Fisher *p* < 0.01 was taken to indicate an excess of overlapping signals. In order to obtain a more robust estimate of the significance of the observed overlap, this was also assessed through permutations. In each permutation cycle, the relation of *p* values to SNPs was randomized within each of the LD-pruned migraine and CAD data sets, and a Fisher *p* for overlap was calculated for each *p* value cutoff. We generated 100,000 permutations to produce an empirical null distribution of *p* values.

In an equivalent manner, secondary analyses were performed for MO (83,373 overlapping SNPs after LD pruning) and MA (88,031 overlapping SNPs after LD pruning).

#### Polygenic risk score analysis.

If shared genetic risk variants are in part or fully responsible for comorbidity between migraine and CAD, we would expect an accumulation of CAD risk alleles in migraineurs. To test this hypothesis, we used the 6 migraine cohorts in which individual-level genotype data were available for analysis (6,350 migraineurs vs 15,069 controls; figure 1). For each migraine case or control, we calculated a CAD polygenic risk score based on a previously published method.^16^ We first generated 3 sets of CAD risk SNPs by selecting SNPs with strong (*p* < 5 × 10^−8^; 149 SNPs), moderate (*p* < 1 × 10^−4^; 1,631 SNPs), or weak (*p* < 1 × 10^−2^; 36,384 SNPs) association to CAD among the 2,342,101 SNPs with information in both migraine and CAD studies. As suggested in the original description of the method,^16^ the analysis was based on non–LD-pruned SNP sets in order to optimize sensitivity. Using each set of CAD risk SNPs, we calculated a per-individual CAD polygenic risk score by summing the number of CAD risk alleles (or expected allele counts for imputed SNPs), each weighted by the log odds ratio from the CAD study. We subsequently assessed whether CAD polygenic risk score was associated with migraine status by applying a logistic regression model of the effect of CAD polygenic risk score (continuous) on migraine status (case, control), adjusted for sex and dummy-coded covariates representing the 6 individual migraine study cohorts.

#### Identifying shared risk loci.

In order to identify shared risk loci between migraine and CAD, we applied a novel method, Cross-Phenotype Spatial Mapping (CPSM; see e-Methods for an overview). This method compares 2 sets of *p* values from GWAS in order to find groups of SNPs at which they are correlated and thus identify shared patterns of association. We applied this method to the 2,342,101 overlapping SNPs from the migraine and CAD studies and selected genomic regions with signal above the 99.95th percentile of 1,000 permutations for further analysis. Potential effects of the shared association loci on regional gene expression (cis effect) were examined using an existing expression quantitative trait locus database from peripheral blood^17^ (e-Methods).

Lastly, we analyzed loci with previously reported genome-wide significant association to migraine or CAD (summarized in the original publications).^12,13^ The lead SNP at each locus was cross-analyzed for association to the other phenotype, Bonferroni correcting for the number of SNPs tested. All 13 of 13 reported migraine loci and 22 of 25 reported CAD loci were available in our data set and could be tested (excluding CAD risk SNPs rs17465637, rs1746048, and rs12413409).

## RESULTS

Comparing nominally significant SNPs from the migraine and CAD GWAS, we found an overlap of association signals in excess of what would be expected by chance (table 1). An overlap of signals was seen for SNPs with *p* values ≤1 × 10^−2^, 1 × 10^−4^, and 1 × 10^−6^. This was supported by permutation testing, which indicated a sharing of association signals at *p* value cutoff 1 × 10^−5^ as well. For reference, the full list of SNPs with association *p* value ≤1 × 10^−2^ to both CAD and migraine is given in table e-3. Secondary analyses by migraine subtype revealed an overlap of association signals between MO and CAD at all *p* value cutoffs (1 × 10^−2^, 1 × 10^−3^, 1 × 10^−4^, 1 × 10^−5^, and 1 × 10^−6^), while no overlap was seen between MA and CAD at any of the *p* value cutoffs. The direction of effect for overlapping association signals did not consistently agree between migraine and CAD, as evidenced by nonsignificant binomial *p* values for concordance (table 1).

**Table 1:**
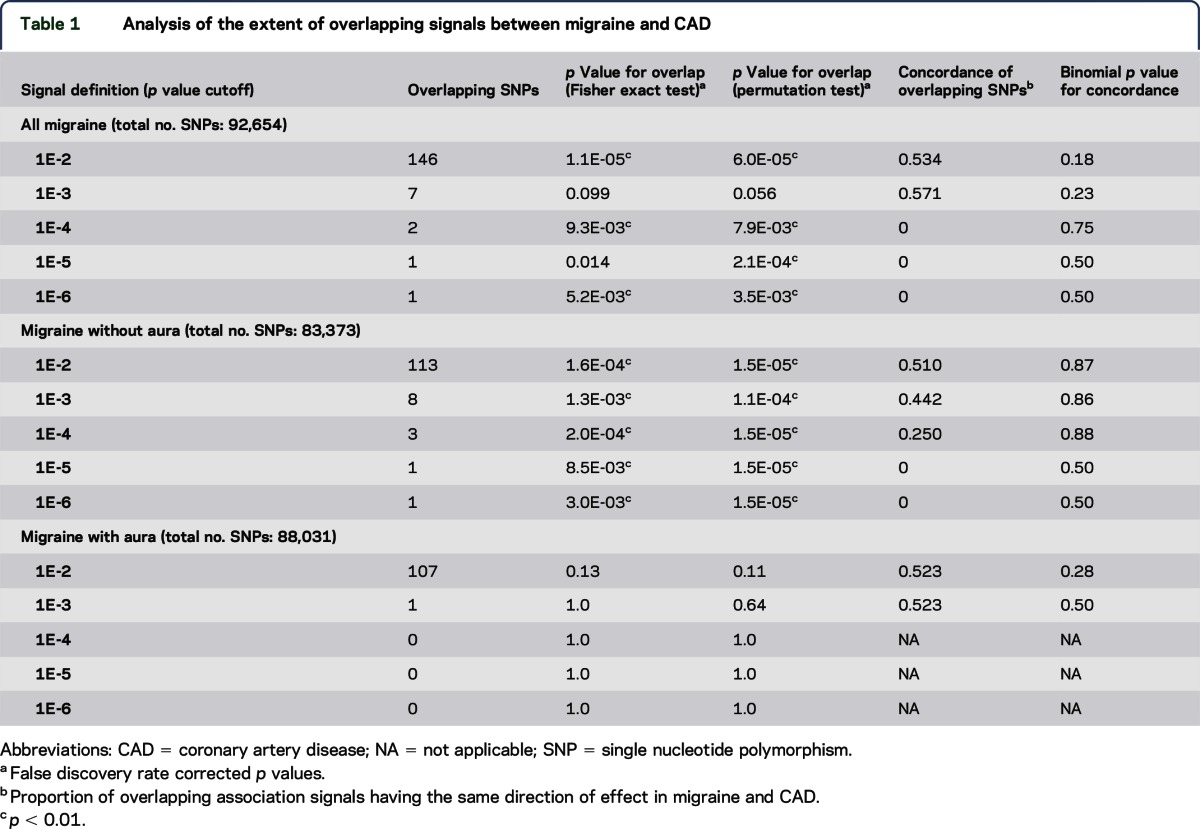
Analysis of the extent of overlapping signals between migraine and CAD

**Figure 2:**
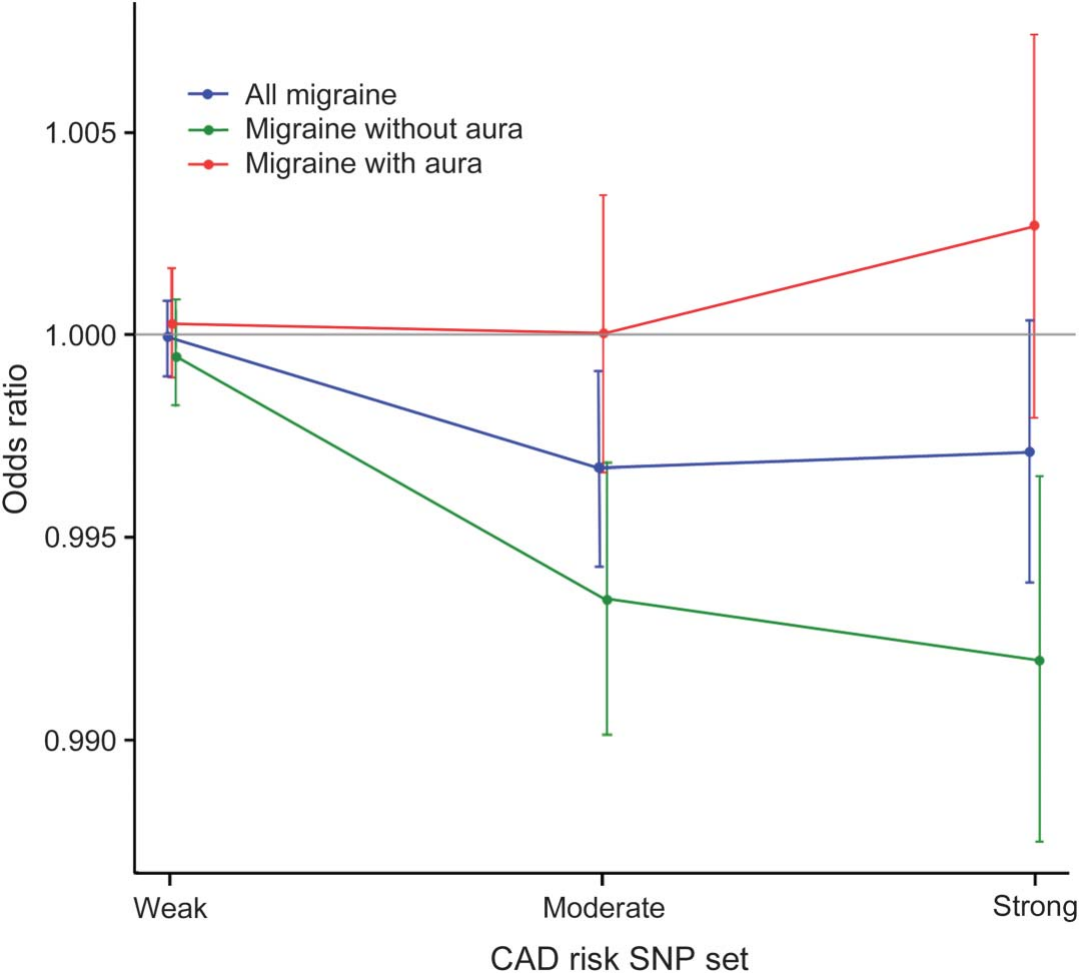
Association between coronary artery disease polygenic risk score and the presence of migraine Results are given as odds ratios with 95% confidence intervals. Separate lines are shown for all migraine (blue), migraine without aura (green), and migraine with aura (red). The coronary artery disease (CAD) polygenic risk score was calculated based on single nucleotide polymorphisms (SNPs) with weak (*p* < 1 × 10^−2^), moderate (*p* < 1 × 10^−4^), or strong (*p* < 5 × 10^−8^) association to CAD in the Coronary ARtery DIsease Genome-Wide Replication And Meta-Analysis study.

To examine this further, the second analysis compared the load of genetic risk variants for CAD between migraineurs and controls, using individual-level data. The results indicated that a high CAD polygenic risk score was associated with a reduced risk of migraine (figure 2, further details in table e-4). For migraine overall, this association was seen for only the moderate CAD risk SNP set (*p* = 0.007). Secondary analyses revealed a similar, but more pronounced, association between CAD polygenic risk score and MO (*p* = 1.5 × 10^−4^ and 5.1 × 10^−4^ for the moderate and strong CAD risk SNP sets, respectively). No association was seen for MA. In the analysis of the weak CAD risk SNP set, there was no association to CAD genetic risk score for either migraine category, indicating that the observed associations were driven by a fairly limited number of loci that are at least moderately associated with CAD. These findings were consistent across men and women (figure e-2) and across individual independent cohorts within the same migraine subtype (figure e-3).

CPSM yielded 16 loci that overlapped between migraine and CAD (table 2; figure e-4). Details of the most significant migraine and CAD SNPs at each locus are given in table e-5. The strongest evidence of shared association was seen at 6p24 (locus no. 1 of table 2), where both CAD and migraine showed genome-wide significant signals within the *PHACTR1* gene (CAD: rs4714955, *p* = 9.8 × 10^−11^; migraine: rs9349379, *p* = 5.9 × 10^−9^). The second strongest overlapping signal was on 17q21 (locus no. 2), where the lead CAD SNP (rs46522, *p* = 2.6 × 10^−7^) was intragenic in *UBE2Z*, whereas the lead migraine SNP (rs11079844, *p* = 3.1 × 10^−5^) was intergenic between *SNF8* and *GIP*. It is interesting that both lead SNPs are in high LD (*r*^2^ > 0.9) with 2 functional variants in *GIP*: Ser103Gly (rs2291725) and a splice site variant (rs2291726) that is predicted to lead to a prematurely truncated transcript^18^ (table e-6). The locus was also found to have a potential effect on the expression level of *UBE2Z* (table e-7). Lead SNPs in 5 loci were in high LD (*r*^2^ > 0.8) with nonsynonymous or splice site variants in nearby genes (table e-6). Ten of the 16 loci showed opposite direction of effect for migraine and CAD. In the secondary analyses, 12 of the 16 lead migraine SNPs had a lower association *p* value in MO than in MA (2-tailed binomial *p* = 0.08), and all 16 SNPs had the same effect direction in each of the 2 migraine subtypes. Local Manhattan plots and covariance plots for the identified loci are given in figure e-4.

**Table 2:**
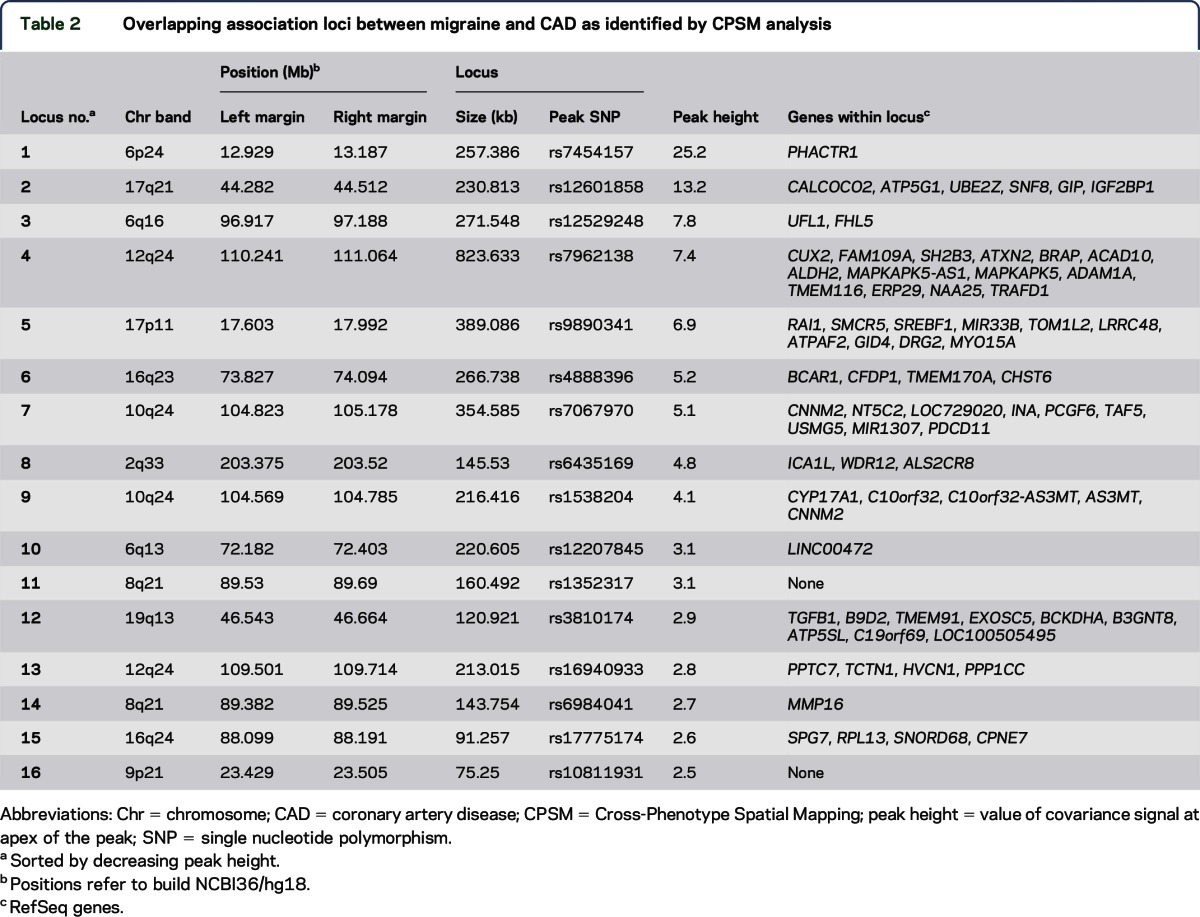
Overlapping association loci between migraine and CAD as identified by CPSM analysis

When considering previously reported risk loci for migraine and CAD, 3 CAD risk SNPs were associated to migraine at study-wide significance, and 2 migraine risk SNPs were associated to CAD (table 3). These correspond to loci no. 1, 2, 3, 11, and 14 as identified by the CPSM method and corroborate the evidence for shared genetic risk at these loci.

**Table 3:**
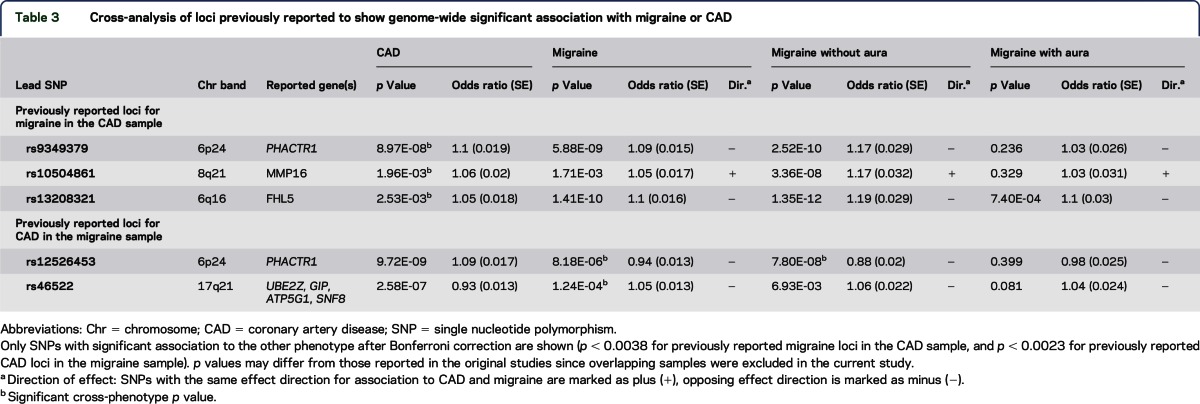
Cross-analysis of loci previously reported to show genome-wide significant association with migraine or CAD

## DISCUSSION

In this study, we used data from 2 recently performed large-scale nonoverlapping GWAS to examine shared genetic risk between migraine and CAD. We found that association signals overlapped in excess of what would be expected by chance. Stratifying by migraine subtype further revealed that MO and MA behaved differently. MO had a genetic overlap with CAD, whereas MA did not. These results are unexpected, given the epidemiologic evidence that comorbidity with CAD is more common in MA than MO. Patients with MA were found to have a 2-fold increased risk for CAD^8,10^ and an increased risk for CAD-related mortality,^9,11^ although one cross-sectional study failed to find an association between CAD and any migraine subtype.^19^ Studies not differentiating on migraine subtype have been less conclusive, with some^8,9,20,21^ but not others^19,22^ indicating an increased risk of CAD related to migraine overall.

For MO, we found a clear overlap of association signals with CAD, whichever *p* value cutoff was used to define signals. Intriguingly, the impact was in the opposite direction, in that patients with MO had a lower load of CAD risk alleles than migraine-free controls. This association seemed to be driven by a limited number of loci. Only a proportion of the included migraine patients were phenotyped in sufficient detail to allow subclassification into MA or MO. When using the considerably larger set of all migraine patients, a similar association was seen as for MO, likely driven by this migraine subtype. While the results suggest that there are shared common risk variants between migraine and CAD, they do not indicate that these variants explain comorbidity between the 2 disorders.

The opposite direction of effect for some of the loci is consistent with a recent GWAS in which the migraine and CAD risk SNP rs9349379 (in *PHACTR1*) was associated with cervical artery dissection, with effect in the same direction as for migraine but opposite of CAD.^23^ Two further migraine SNPs showed evidence of association to cervical artery dissection with the same effect direction as for migraine (rs11172113 in *LRP1* and rs13208321 in *FHL5*, the latter identified as locus 3 in the current study) but opposite direction for CAD.

The significant sharing of risk loci between migraine and CAD may reflect that they involve some of the same biological processes. Experimental studies will be needed to clarify this and whether the shared risk loci can give information on vascular mechanisms involved in migraine pathogenesis.

The lack of overlapping association signals between MA and CAD may indicate that the 2 disorders have separate and nonrelated genetic backgrounds. However, it may also result from insufficient power to detect shared common genetic risk factors for this migraine subtype. This is consistent with the relative failure so far in identifying common risk variants for MA; despite at least as high heritability and comparable study sample sizes, only one genome-wide significant locus has been identified for MA, compared to 9 for MO.^12,24–27^ It is possible that MA is a more heterogeneous disorder or is influenced by rare and low-frequency variants not captured by current imputation panels.^12,28^ Larger studies that also interrogate rare variants will be needed to determine the genetic basis of MA and its potential overlap with cardiovascular disease.

Six of the overlapping loci have previously been associated with CAD at genome-wide significant levels (loci 1, 2, 4, 7, 8, and 9 of table 2),^13,29^ and 2 with migraine (loci 1 and 3).^12,25^ The strongest overlapping region (locus 1) is entirely intragenic in *PHACTR1* (which encodes phosphatase and actin regulator 1 protein). This locus is associated with both migraine and CAD at genome-wide significant levels in the current and previous studies^13,25,30^ and has also been associated with coronary artery calcification and stroke.^31,32^ *PHACTR1* is highly expressed in the brain, and its transcript is an important regulator of synaptic activity and dendritic morphology through the control of protein phosphatase 1 and actin binding.^33^ More recently, *PHACTR1* has been identified as a key regulator of endothelial function, including endothelial cell survival and angiogenesis,^34^ and it is associated with altered vasomotor tone.^35^ Both endothelial and vasomotor dysfunctions have been implicated in migraine,^36,37^ and this locus offers a potential focus for future studies. Alternatively, the pleiotropic effects of this gene on both synaptic and vascular functions may give rise to independent causal pathways for the 2 disorders.

The second strongest overlapping region (locus 2) is a previously identified risk locus for CAD.^13^ The lead CAD (rs46522) and migraine variants (rs11079844) are in strong LD (*r*^2^ = 0.94), and both are in strong LD (*r*^2^ > 0.90) with 2 potentially functional variants in *GIP* (which encodes gastric inhibitory polypeptide). *GIP* regulates glucose-induced insulin release from pancreatic β-cells and helps resensitize the insulin response.^38^ It is also expressed in the brain, where it may be involved in proliferation of neuronal progenitor cells.^39^ Whether *GIP* is involved in the observed tendency for insulin resistance and metabolic syndrome in migraineurs should be investigated.^40^

Strengths of this study include the use of large-scale nonoverlapping GWAS of migraine and CAD, stringent quality control measures, and sufficiently rich phenotyping to allow secondary analyses of the 2 migraine subtypes. Nevertheless, some limitations should also be acknowledged. First, only summary statistics and not individual-level genotype data were available for the majority of the samples included in this study. Second, in each included cohort, phenotype information was available on only migraine or CAD, not both. This prevented us from performing more in-depth analyses, including analysis for potential gene-gene interactions or identification of CAD risk loci specific to migraineurs. Third, considerable effort was devoted to the careful avoidance of shared controls between studies, and stringent quality control measures within each data set were enforced to reduce the risk of spurious effects resulting from biases within the data sets. Nevertheless, we cannot rule out subtle biases that could affect the current results. Two such concerns are the effects of migraine on survival and the possibility that migraineurs may be more likely to seek medical treatment and therefore be under closer surveillance with regards to other disorders. Future efforts should aim to replicate these findings in sufficiently large prospective data sets where both phenotypes are measured in the same individuals.

Our study provides novel insights into the relationship between migraine and CAD. Intriguingly, and unexpectedly, there was no genetic overlap between MA and CAD, for which epidemiologic studies suggest comorbidity, but there was compelling evidence for a genetic overlap between MO and CAD, where the impact of risk variants overall was in opposite direction for the 2 disorders. The results do not demonstrate that shared common genetic risk factors drive comorbidity between the 2 disorders. However, dissecting the mechanisms underlying the shared risk loci may improve our understanding of both disorders.

## Supplementary Material

Data Supplement

Coinvestigators

## GLOSSARY

CAD: coronary artery disease
CARDIoGRAM: Coronary ARtery DIsease Genome-Wide Replication And Meta-Analysis
CPSM: Cross-Phenotype Spatial Mapping
GWAS: genome-wide association studies
IHGC: International Headache Genetics Consortium
LD: linkage disequilibrium
MA: migraine with aura
MO: migraine without aura
SNP: single nucleotide polymorphism
