# Supplementary material for: Genetic analysis for a shared biological basis between migraine and coronary artery disease
Source: Neurol Genet. 2015 Jul 2;1(1):e10. doi: 10.1212/NXG.0000000000000010 (PMC4821079; doi:10.1212/NXG.0000000000000010)
Supplement: Data Supplement [file supp_1_1_e10__index.html]

Data Supplement 

# Genetic analysis for a shared biological basis between migraine and coronary artery disease

## Data Supplement

**Files in this Data Supplement:**

- e-Methods - PDF
- Figure e-1 - e-4 - PDF
- Table e-1 - e-7 - PDF
