# Supplementary material for: Genetic analysis for a shared biological basis between migraine and coronary artery disease
Source: Neurol Genet. 2015 Jul 2;1(1):e10. doi: 10.1212/NXG.0000000000000010 (PMC4821079; doi:10.1212/NXG.0000000000000010)
Supplement: Data Supplement [file supp_1.1.e10_e-Methods.pdf]

Supplemental Methods

Cohorts and sample collection

The International Headache Genetics Consortium (IHGC) study collected 23 285 cases with migraine and 95 425 controls from 29 studies for a meta-analysis of genome-wide association data.[1] For a subset of the cohorts sufficiently detailed phenotype information was available to allow subclassification into either of the two migraine subtypes, migraine with aura (5 118 cases versus 74 239 controls) or migraine without aura (7 107 cases versus 69 427 controls).[2] The CARDIoGRAM study combined data from 14 GWAS studies of CAD for a total of 22 233 cases versus 64 762 controls. Three study cohorts were part of both the IHGC and CARDIoGRAM studies (deCODE, 1958 Birth Cohort and KORA). To assure independence between the migraine and CAD datasets we reperformed the IHGC migraine meta-analysis without deCODE and 1958 Birth Cohort, and the CARDIoGRAM CAD meta-analysis without GERMIFS III (KORA), resulting in two datasets without overlapping samples (19 981 migraine cases versus 56 667 controls, and 21 076 CAD cases versus 63 014 controls). Descriptions of the included cohorts, and criteria for diagnosing migraine or CAD in each study, are given in **Tables e1 and e2**. In addition to using summary statistics (*P* value, effect allele, effect size for SNPs) from these studies, we also used individual-level genotype data from the six migraine cohorts where such data were available: German MA (997 cases, 1105 controls), German MO (1208 cases, 2564 controls), LUMINA MA (820 cases, 4774 controls), LUMINA MO (1118 cases, 2016 controls), Finnish MA (1032 cases, 3513 controls) and the HUNT Study, Norway (MO cases only; 1175 cases, 1097 controls).

Genome-wide association studies

For a complete description see the original publications of the CARDIoGRAM[3] and IHGC migraine studies.[1] In the IHGC migraine study genome-wide SNP genotyping was performed independently in each cohort with the use of various standard genotyping technologies, and imputed for each study with reference to HapMap release 21 or 22 CEU phased genotypes.[4] Each study contributed summary statistic data from an association analysis performed using a frequentist additive model based on an expected allelic dosage model for SNP markers, adjusting for gender. SNPs were filtered on per-study level based on inclusion criteria of MAF>0.1% and imputation quality measures of $I_A > 0.6$ (IMPUTE 2[5]) or $r^2 > 0.3$ (MACH[6]). In the meta-analysis performed for migraine in the current study, combined association data for about 2.5 million imputed and genotyped autosomal SNPs were analyzed in a fixed-effects model using GWAMA. At this stage, SNPs with a heterogeneity coefficient I^2 exceeding 75% or presence in less than five studies were filtered out. In the meta-analysis, there was little evidence of population stratification at the study level (each genomic inflation factor $\lambda \leq 1.1$), though moderate inflation was observed at the meta-analysis level ($\lambda = 1.13$).

In the CARDIoGRAM study genotyping was performed independently in each cohort with the use of Affymetrix or Illumina platforms, and individual studies were imputed with reference to HapMap release 21 or 22. Association analysis for each study was performed using a log-additive model frequentist test adjusting for age and gender and taking into account the uncertainty of possibly imputed genotypes. Quality control was performed centrally according to previously agreed criteria including check of consistency of the given alleles across all studies, quality of the imputation, deviation from Hardy-Weinberg equilibrium in the controls, minor allele frequency, and call rate. In the CAD meta-analysis our default was a fixed-effect model with inverse variance weighting, but as in the original CARDIoGRAM study, where heterogeneity was present (P for Cochran’s Q <0.01) we adopted and reported a random-effects model (DerSimonian-Laird) for that SNP. There was

little evidence of population stratification at the study level,[3] though moderate inflation was observed at the meta-analysis level in the current study ($\lambda = 1.14$).

Allele specific frequencies were compared between the two datasets (migraine and CAD), to make sure that analyses were not biased by systematic allele flips. Manhattan plots for the migraine and CAD genome-wide association analyses used in the current study are given in **Figure e4**.

Cross-phenotype spatial mapping

Our cross-phenotype spatial mapping method (fully described in an upcoming manuscript) identifies genomic windows exhibiting similar association patterns across two phenotypes using a signal processing approach. We compute Pearson's covariance between *P* values from two traits across a 60 kb sliding window. In each iteration, the window slides to the next SNP so we obtain a covariance coefficient for each SNP in the analysis. We then detect peaks of signal across the genome in the covariance trace. The signal s_n for a given SNP with index *n*, position b_n (base pairs), and association *P* values $p_{1,n}, p_{2,n}$ for two phenotypes is calculated as follows:

$$x = \left[-\log_{10} p_{1,j}, \ldots, -\log_{10} p_{1,k}\right] \quad (1)$$

$$y = \left[-\log_{10} p_{2,j}, \ldots, -\log_{10} p_{2,k}\right]. \quad (2)$$

$$w = \left[1 - \frac{|b_j - b_n|}{b_k - b_j}, \ldots, 1 - \frac{|b_k - b_n|}{b_k - b_j}\right] \quad (3)$$

$$w' = \frac{1}{\sum_{i=j}^{k} \omega_i} \cdot w \quad (4)$$

$$s_n = \frac{\sum_{i=j}^{k} \omega'_i x_i y_i}{1 - \sum_{i=j}^{k} \omega'^2_i} \tag{5}$$

Where each $b_i \in b_{j,}, \ldots, b_k$ is the position of SNP *i* within the window of SNPs $j,\cdots,k$ containing SNP *n*. For a given window size *d* (base pairs), the window of SNPs $j,\cdots,k$ is defined such that *j* is the smallest SNP index where $b_n - b_j \leq \frac{d}{2}$ and *k* is the largest SNP index where $b_k - b_n \leq \frac{d}{2}$.

After constructing the CPSM signal for all SNPs, we correct for strong associations present in only a single phenotype by permuting the association *P* values for one phenotype 1,000 times while holding the other phenotype constant, and recalculating CPSM. From the total set of 2,000 permutation signals (1,000 per phenotype), we take the upper 0.95 quantile at each SNP as the background signal threshold and subtract it from the observed covariance as a correction. We then detect regions of shared association as peaks above the 99.95th percentile of the covariance signal.

Expression quantitative trait locus (eQTL) analysis

In order to identify eQTLs that could explain the association signals at overlapping loci, we used a previously published eQTL dataset consisting of 3 754 samples from peripheral venous blood.[7] To test for overlapping eQTLs, we first created a set of markers at each of the 16 overlapping CPSM loci that could be assumed to be credibly causal, using the method defined in a previous paper[8] and association P values from the migraine dataset. For the most significant eQTLs ($p<1x10^{-4}$) that were found to any genes within a 1Mb window of each locus, we created a set of eQTL loci where each locus was defined as the physical region containing all SNPs in LD (r2>0.6) with the top eQTL SNP to a particular gene. We then

further refined each eQTL locus to contain only a credible set of markers for each eQTL effect., and finally tested for significant correlation between the z-scores in each migraine credible set compared to each overlapping eQTL credible set. Correlation was measured using Pearson's correlation coefficient and significance assessed by a two-tailed t-test with n-2 degrees of freedom, where n is the number of SNPs overlapping between the two credible sets. Significant correlation between a migraine credible set and an eQTL credible set after Bonferroni correction was taken as evidence of the migraine locus tagging a real eQTL.

References

1. Anttila V, Winsvold BS, Gormley P, et al. Genome-wide meta-analysis identifies new susceptibility loci for migraine. Nature genetics 2013;45:912-917.
2. Headache Classification Committee of the International Headache Society (IHS). The International Classification of Headache Disorders, 3rd edition (beta version). Cephalalgia : an international journal of headache 2013;33:629-808.
3. Schunkert H, Konig IR, Kathiresan S, et al. Large-scale association analysis identifies 13 new susceptibility loci for coronary artery disease. Nature genetics 2011;43:333-338.
4. Frazer KA, Ballinger DG, Cox DR, et al. A second generation human haplotype map of over 3.1 million SNPs. Nature 2007;449:851-861.
5. Howie BN, Donnelly P, Marchini J. A flexible and accurate genotype imputation method for the next generation of genome-wide association studies. PLoS genetics 2009;5:e1000529.
6. Li Y, Willer CJ, Ding J, Scheet P, Abecasis GR. MaCH: using sequence and genotype data to estimate haplotypes and unobserved genotypes. Genetic epidemiology 2010;34:816-834.
7. Wright FA, Sullivan PF, Brooks AI, et al. Heritability and genomics of gene expression in peripheral blood. Nature genetics 2014;46:430-437.
8. Maller JB, McVean G, Byrnes J, et al. Bayesian refinement of association signals for 14 loci in 3 common diseases. Nature genetics 2012;44:1294-1301.
