# Supplementary material for: Genetic analysis for a shared biological basis between migraine and coronary artery disease
Source: Neurol Genet. 2015 Jul 2;1(1):e10. doi: 10.1212/NXG.0000000000000010 (PMC4821079; doi:10.1212/NXG.0000000000000010)
Supplement: Data Supplement [file supp_1.1.e10_Figures_e-1-e-4.pdf]

Figure e1. Manhattan plots for the meta-analyses of genome-wide association data used in the current study.

A)

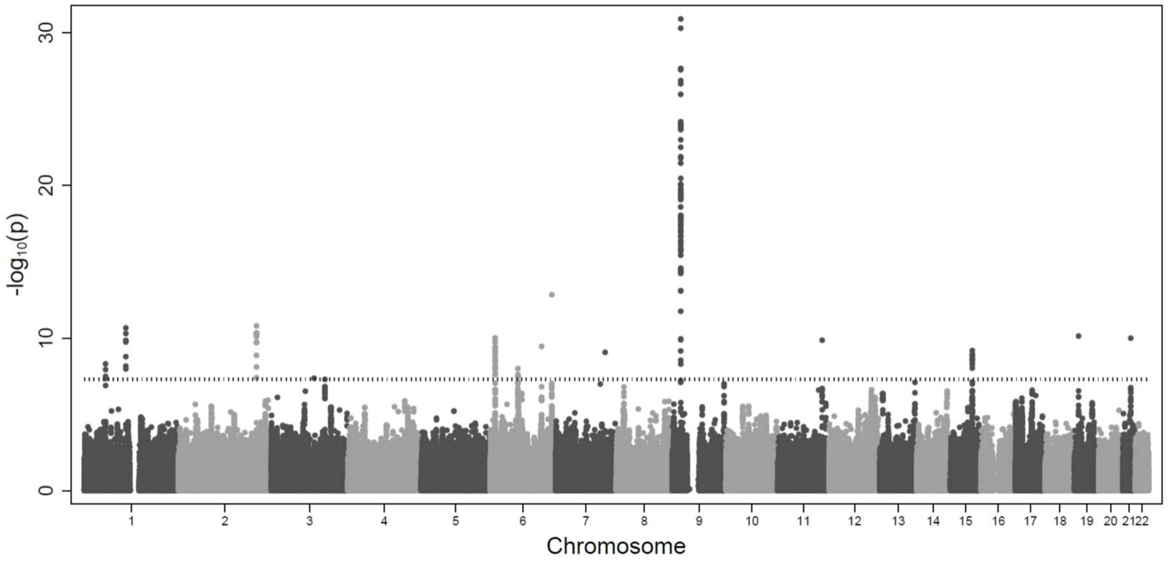

B)

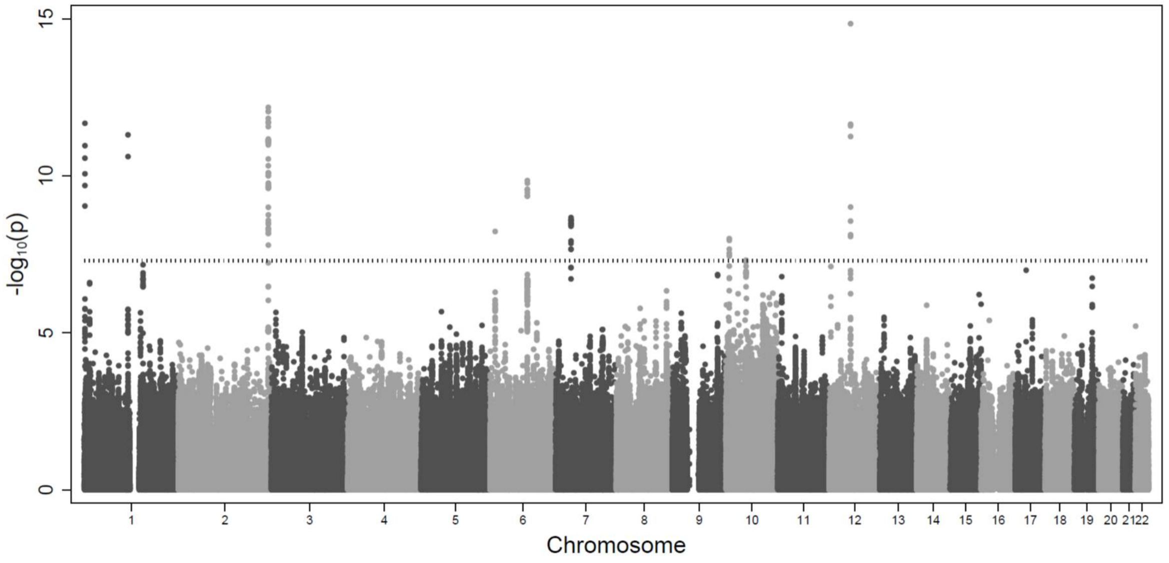

C)

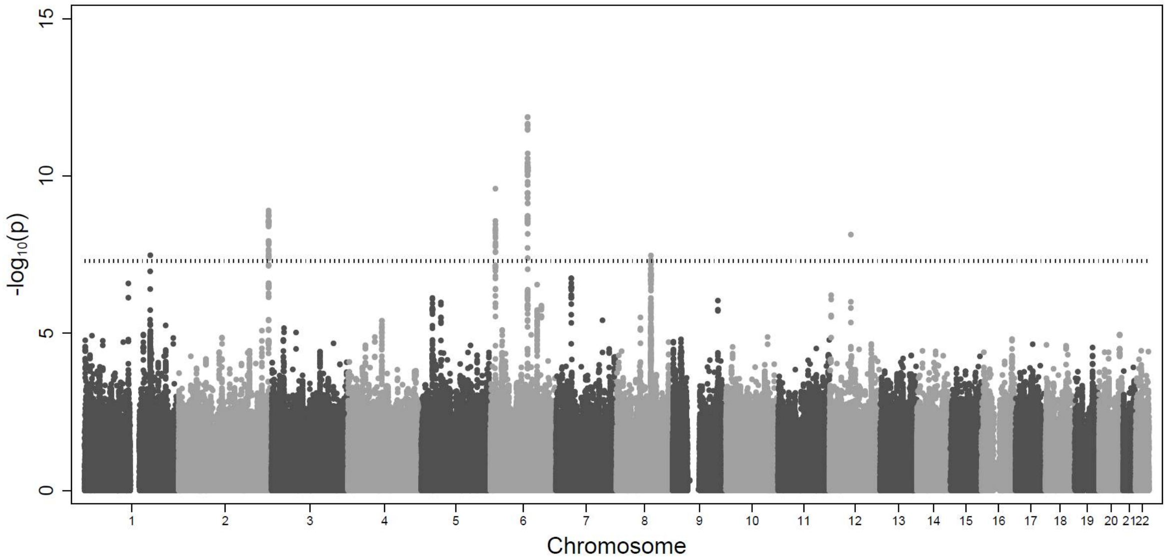

D)

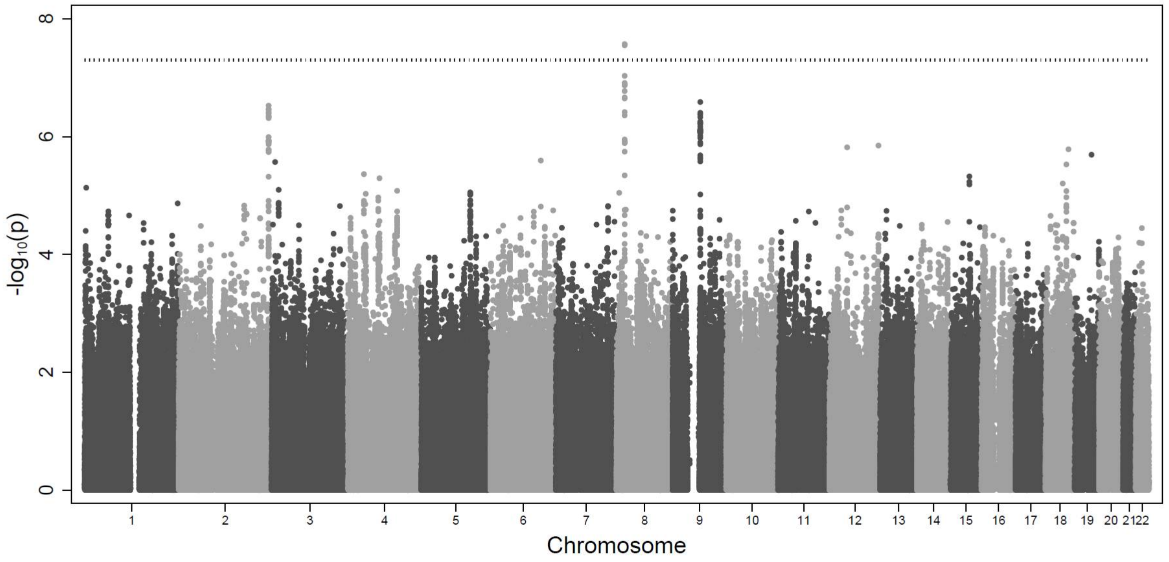

Grey horizontal line indicates genome-wide significance level ($P < 5 \times 10^{-8}$). Separate plots are shown for A) Coronary artery disease (21 076 cases versus 63 014 controls), B) Migraine (19 981 cases versus 56 667 controls), C) Migraine subanalysis: Migraine without aura (6 413 cases versus 32 745 controls), D) Migraine subanalysis: Migraine with aura (4 940 cases versus 37 557 controls).

Figure e2. Association between coronary artery disease polygenic risk score and the presence of migraine, stratified by gender.

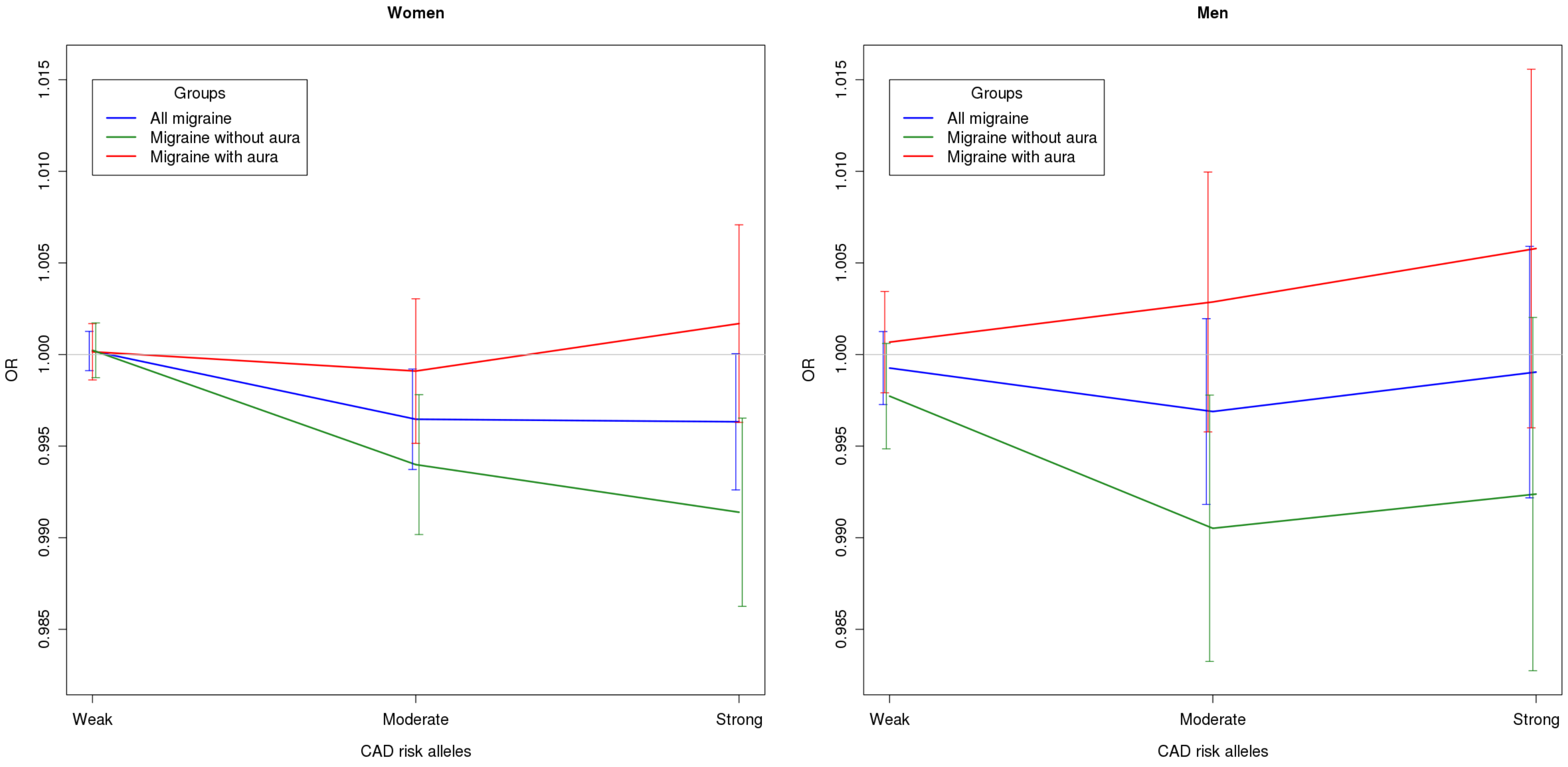

CAD = coronary artery disease.

Results are given as odds ratios (OR) with 95% confidence intervals. Separate plots are shown for women (6 395 females cases versus 9 468 female controls) and men (1 563 male cases versus 6 698 male controls). Separate lines are given for all migraine (blue), migraine without aura (green), and migraine with aura (red). The CAD polygenic risk score was calculated based on SNPs with either weak ($P<1 \times 10^{-2}$), moderate ($P<1 \times 10^{-4}$) or strong ($P<5 \times 10^{-8}$) association to CAD in the CARDIoGRAM study.

Figure e3. Association between coronary artery disease polygenic risk score and the presence of migraine, stratified by migraine aura status.

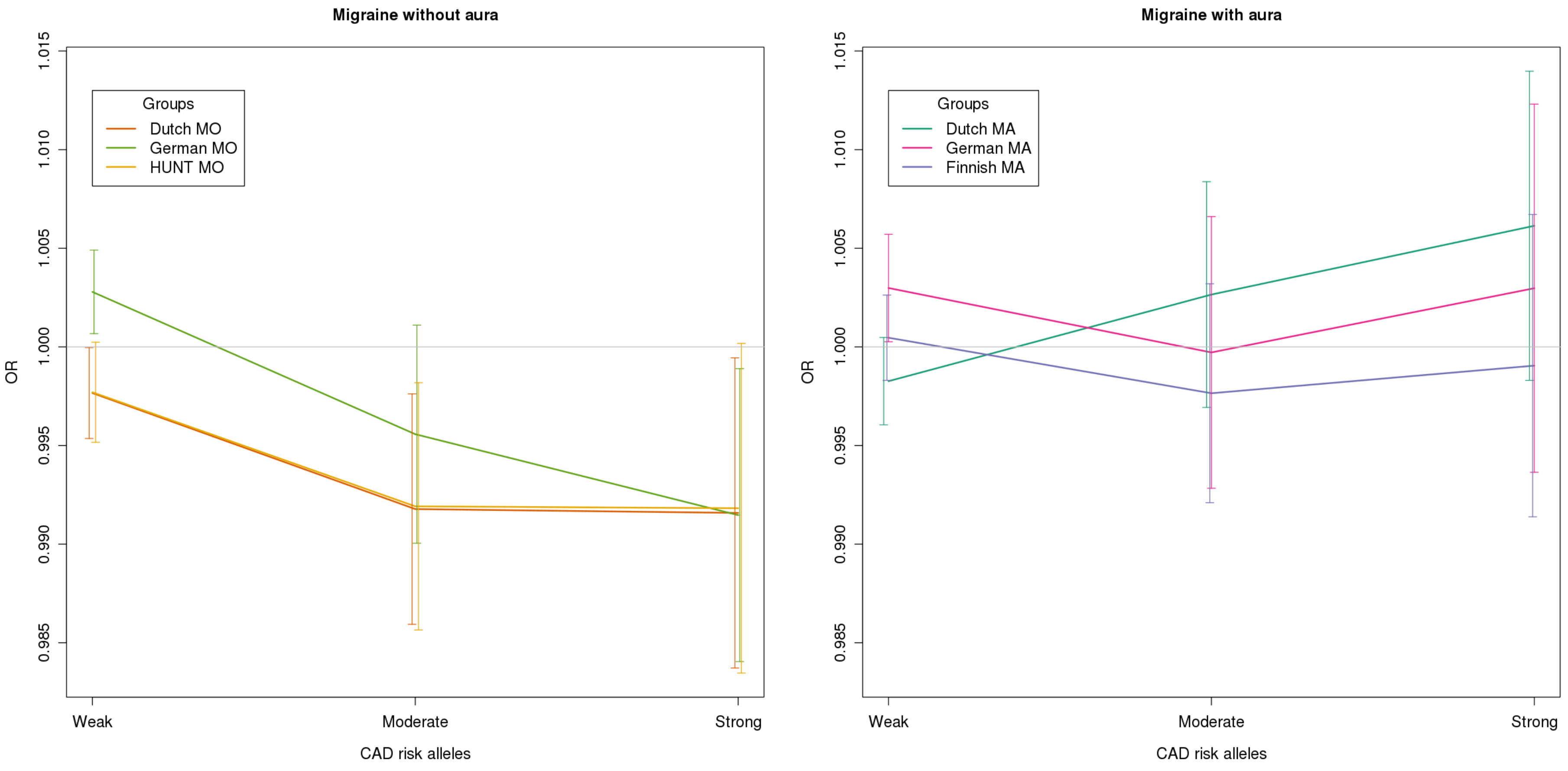

CAD = coronary artery disease.

Results are given as odds ratios (OR) with 95% confidence intervals. Separate plots are shown for migraine without aura (3 501 cases versus 5 677 controls) and migraine with aura (2 849 cases versus 9 392 controls). Separate lines are given for each cohort. The CAD polygenic risk score was calculated based on SNPs with either weak ($P<1 \times 10^{-2}$), moderate ($P<1 \times 10^{-4}$) or strong ($P<5 \times 10^{-8}$) association to CAD in the CARDIoGRAM study.

Figure e4: Local Manhattan plots and covariance signals at the overlapping loci identified by cross-phenotype spatial mapping.

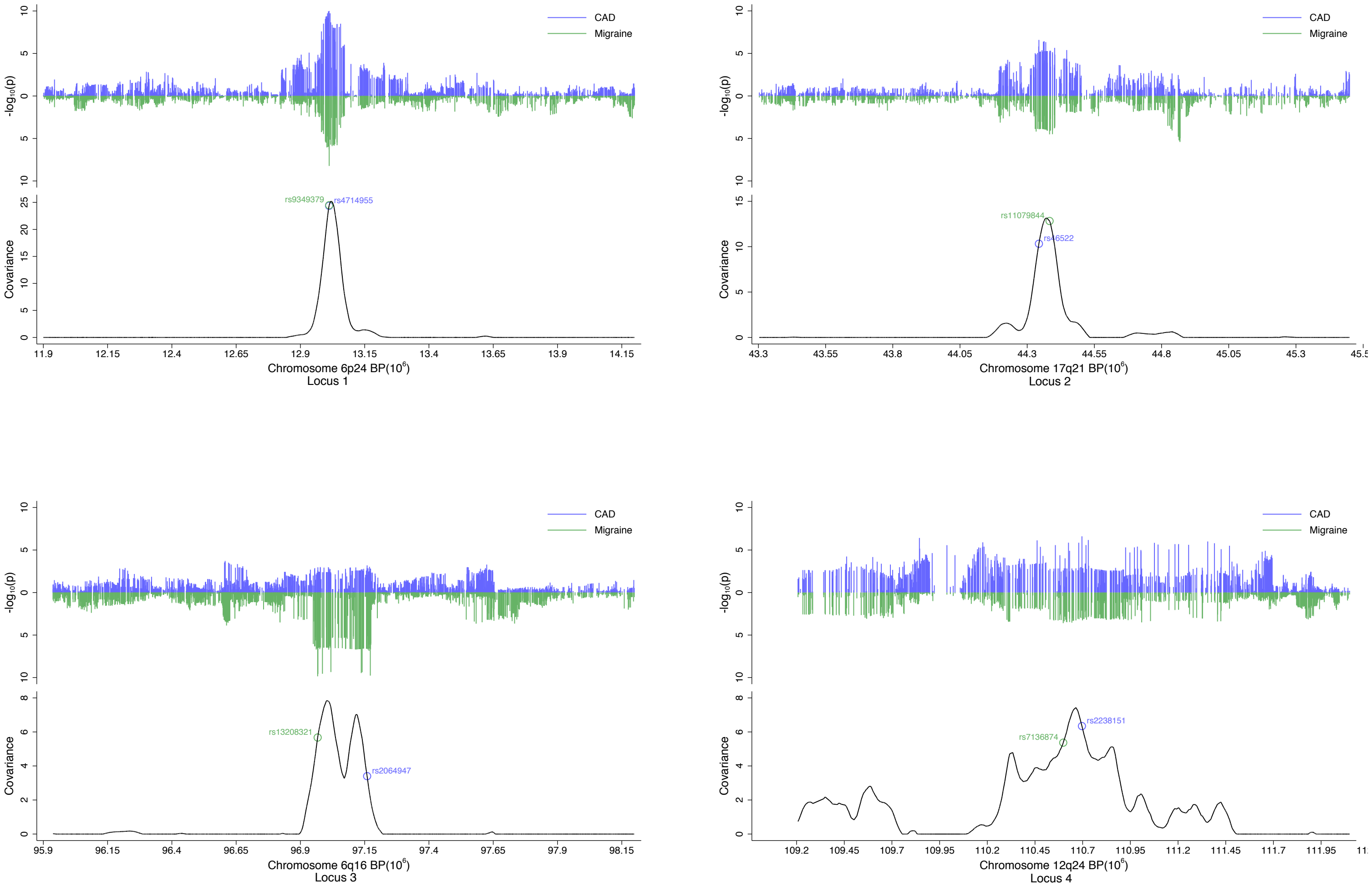

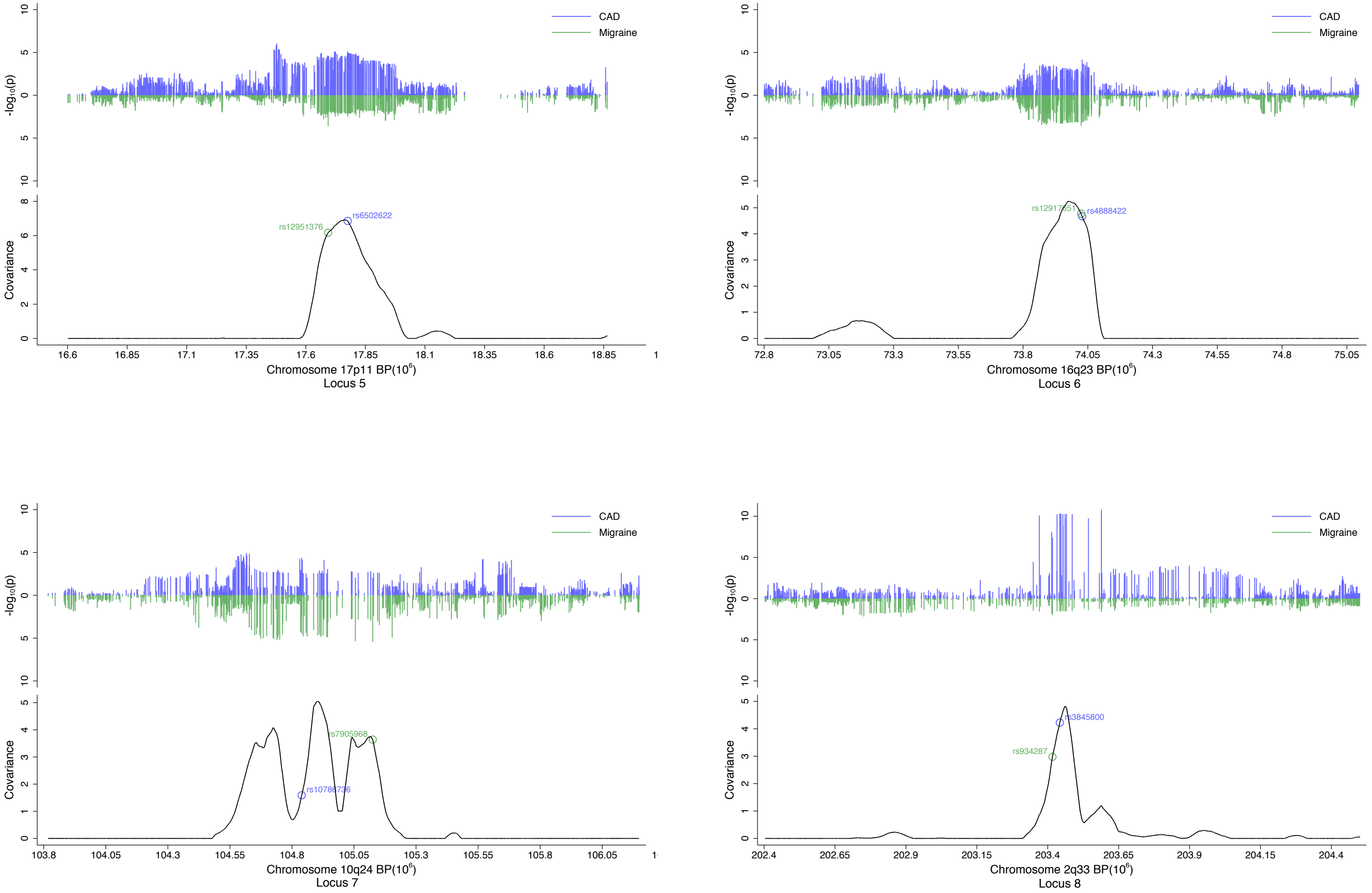
CAD
Migraine
-log10(p)
Covariance
rs12951376
rs6502622
Chromosome 17p11 BP(10^6)
Locus 5
rs4888422
Chromosome 16q23 BP(10^6)
Locus 6
rs7905968
Chromosome 10q24 BP(10^6)
Locus 7
rs3845800
rs934287
Chromosome 2q33 BP(10^6)
Locus 8

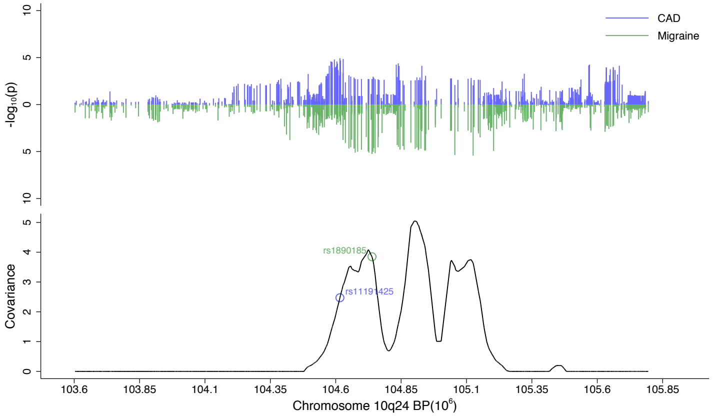

Locus 9

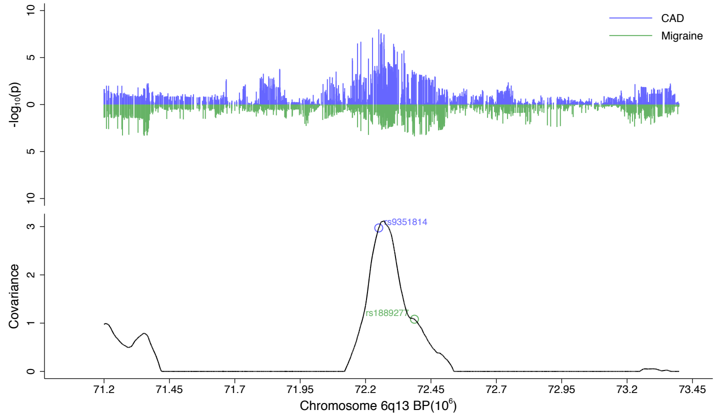

Locus 10

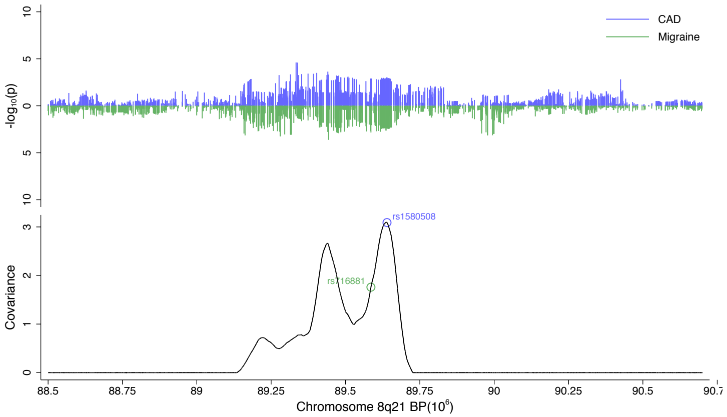

Locus 11

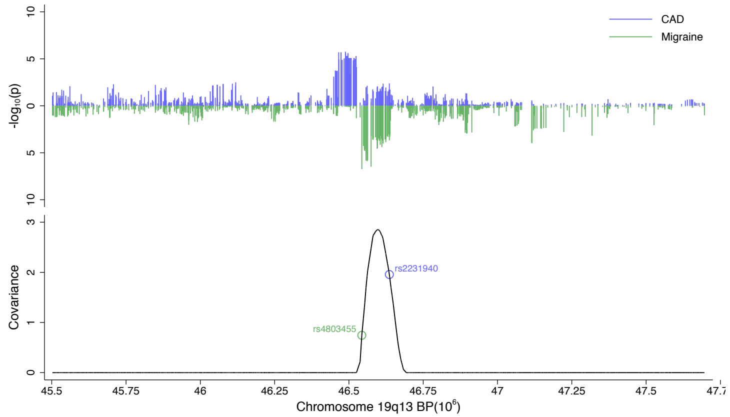

Locus 12

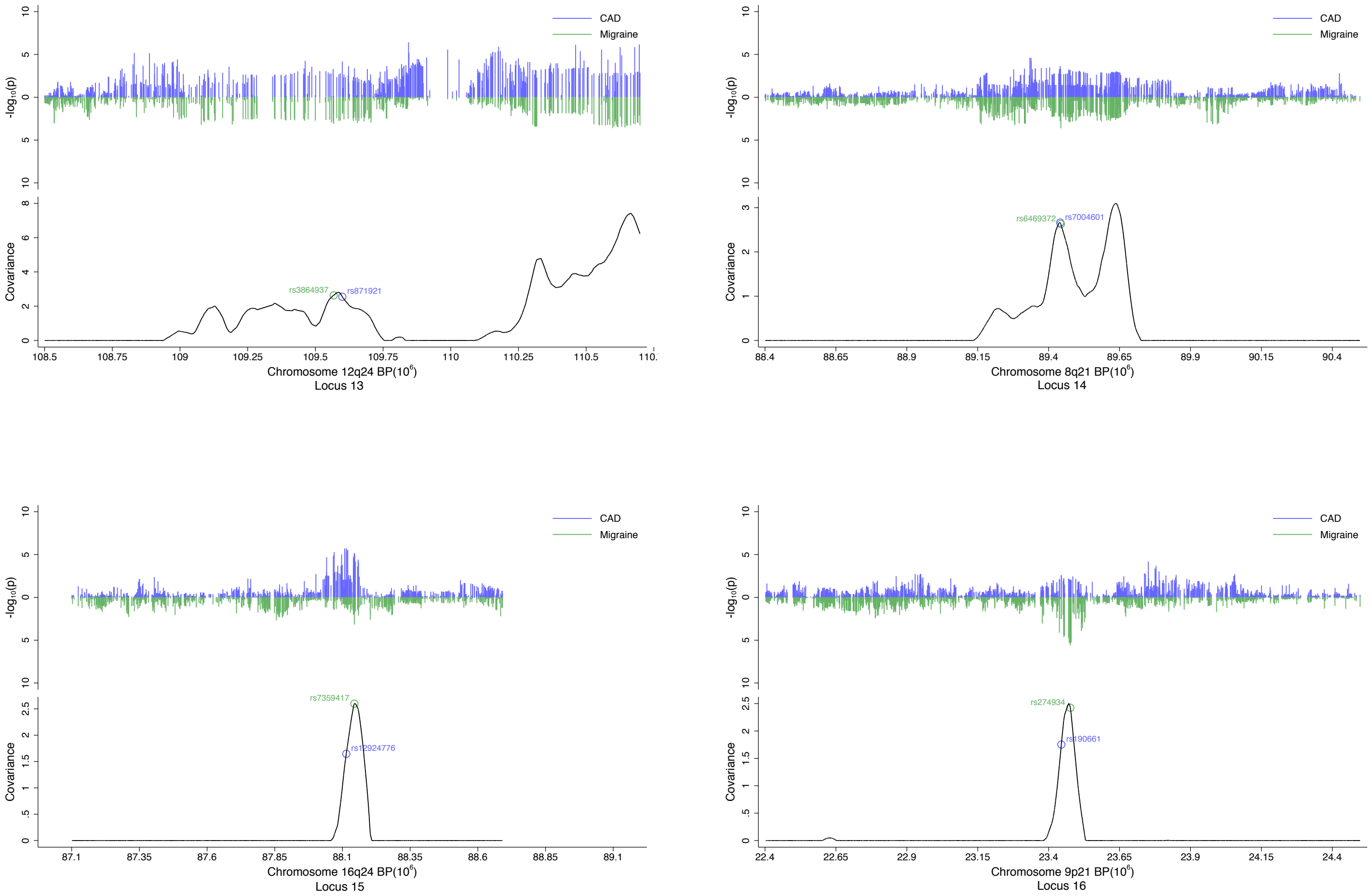

CAD = coronary artery disease.

For each locus $-\log_{10} P$ values are given for both CAD (blue) and migraine (green). In the covariance plots, circles mark the lead SNPs for CAD (blue circle) and migraine (green circle).
