# Supplementary material for: Genetic analysis for a shared biological basis between migraine and coronary artery disease
Source: Neurol Genet. 2015 Jul 2;1(1):e10. doi: 10.1212/NXG.0000000000000010 (PMC4821079; doi:10.1212/NXG.0000000000000010)
Supplement: Data Supplement [file supp_1.1.e10_Tables_e-1-e-7.pdf]

Table e1. Description of migraine cohorts included in the current study.

Study	Cases/ Controls	Migraine %	Female % of cases	Female % of controls	Ethnicity	Migraine definition	Control definition	Ref study PMID
**ALSPAC**	3134/5103	38.0	100.0	100.0	European, British	Self-reported migraine, current or prior	No migraine or use of migraine medications; Pop.	22507742
**Australia ATM**	1683/2383	41.4	72.3	48.6	European descent	Modified ICHD-II criteria, current migraine	Pop.	20303062, 18676988
**ERF**	330/1216	21.3	75.5	50.6	European, Dutch	Full ICHD-II criteria, current migraine	No migraine	20071666
**Finnish MA**	1032/3513	22.7	80.2	52.6	European, Finnish			
MA cases	1032/0					ICHD-II		11509082; 20802479
Health 2000	0/1862						Mig-free	20532202
Helsinki Birth Cohort	0/1651						Pop.	16251536
**FinnTwin**	189/580	28.5	63.5	46.0	European, Finnish	Self-reported migraine, current or prior	No migraine, use of migraine medication or severe recurrent headaches	12537859, 12537860, 20953688
**German MA**	997/1105	47.4	81.1	60.8				
MA cases	997/0				European, German	ICHD-II		20802479
PopGen	0/661				European, German		Pop.	16490960
Illumina iControlDB	0/444				MDS-filtered Caucasian		Pop.	-
**German MO**	1208/2564	32.0	87.0	55.1	European, German			
MO cases	1208/0					ICHD-II		22683712
GSK	0/861						Pop.	19107115
KORA	0/834						Pop.	16032514
MPIPSYKL	0/489						Pop.	-
HNR	0/380						Pop.	12177636
**HUNT**	1608/1097	59.4	74.9	75.0	European, Norwegian	Self-reported migraine or fulfilling Modified	No migraine; Pop.	10999674

						ICHD-II criteria, current migraine		
**LUMINA MA**	820/4774	14.7	82.2	58.6	European, Dutch			
MA cases	820/0					ICHD-II		21914734; 20802479
Rotterdam I	0/4774						Pop.	21877163
**LUMINA MO**	1118/2016	35.7	85.8	54.2	European, Dutch			
MO cases	1118/0					ICHD-II		21914734; 22683712
Rotterdam II	0/2016						Pop.	21877163
**NTR**	265/2128	11.1	85.3	55.2	European, Dutch	Modified ICHD-II criteria, current migraine	No migraine or severe recurrent headache	17254420, 16611468
**NESDA**	17/132	11.4	82.4	56.1	European, Dutch	Modified ICHD-II criteria, current migraine	No migraine or severe recurrent headache	18763692, 20713558
**NFBC**	757/4399	14.7	76.5	48.2	European, Finnish	Self-reported migraine, current or prior	No migraine	4911003
**Rotterdam III**	351/1647	17.6	77.5	51.2	European, Dutch	Modified ICHD-II criteria, current migraine	No migraine	21877163
**TWINS UK**	972/3837	20.2	96.2	89.8	European, British	Self-reported migraine or fulfilling Modified ICHD-II criteria, current or prior migraine	No migraine	22253318
**WGHS**	5122/18108	22.0	100.0	100.0	European descent	Self-reported migraine or fulfilling Modified ICHD-II criteria, current or prior migraine	No migraine	18263651
**Young Finns**	378/2065	15.5	79.6	49.3	European, Finnish	Full ICHD-II criteria, current migraine	No migraine	18263651

The indented rows describe studies that were combined together to generate the set named on the first line of the set.

Where more than one control definition was used, control groups are listed in decreasing size.

ICHD-II = cases fulfill the International Classification of Headache Disorders, 2nd edition definition for current or past migraine; Pop. = Unscreened population-matched population-based sample; Mig-free = migraine-free population-matched population-based sample; PMID = PubMed ID; MA = migraine with aura; MO = migraine without aura.

Table e2. Description of coronary artery disease cohorts included in the current study.

Study	Cases/ Controls	Myocardial infarction %	Female % of cases	Female % of controls	M (SD) Age cases/controls	CAD definition	Control definition	Ref study PMID
**ADVANCE**	278/312	50.4	57.9	59.0	45.8 (6.2)/ 45.3 (5.7)	Clinical non fatal CAD men ≤45 yrs, women ≤55 yrs) including AMI (enzymes), typical angina with ≥1 artery with >50% stenosis, positive non invasive test, or PCI or CABG	No history of clinical CAD, CVA, or PAD	18443000
**CADomics**	2078/2952	58.3	21.9	50.5	60.8 (10.1)/ 55.3 (10.8)	CAD: >50% stenosis in 1 major coronary artery and/or MI based on ECG and enzyme	Population sample with no history of MI	-
**CHARGE**	2287/22024	48.0	33.4	59.6	28.1 (7.4)/ 27.5 (8.0)	CHD: definite or probably MI, PTCA or CABG, or ECG MI	None of the conditions that define CAD	20031568
**deCODE CAD**	6640/27611	54.7	36.3	61.9	74.8 (11.8)/ 53.7 (21.5)	MI: MONICA criteria (<75 yrs) or discharge diagnosis of MI; CAD: PCI or participation in CVD genetics program with self-report or CABG or PCI, or discharge diagnosis of angina pectoris, MI or chronic heart disease	Population sample	17478679
**GERMIFS I**	884/1604	100.0	49.4	50.8	50.2 (7.8)/ 27.7 (4.5)*	MI (<65 yrs) with >1 1st degree sibling with severe CAD (PTCA; MI; CABG)	Population sample	17634449
**GERMIFS II**	1222/1287	100.0	33.1	48.3	51.4 (7.5)/ 51.2 (11.9)*	MI (<60 yrs); 59.4% with family history of CAD	Population sample	19198612
**LURIC/**	652/213	71.9	20.3	46.0	61.0 (11.8)/	Symptoms of angina	No coronary lesions or	11258203

**AtheroRemo 1**					58.3 (12.1)	pectoris, NSTEMI, STEMI, or >50% coronary stenosis	minor stenoses (<20%)	
**LURIC/ AtheroRemo 2**	486/296	79.0	23.4	48.6	63.7 (9.4)/ 56.4 (12.7)	Symptoms of angina pectoris, NSTEMI, STEMI, or >50% coronary stenosis	No coronary lesions or minor stenoses (<20%)	11258203
**MedStar**	874/447†	48.1	33.0	54.6	48.9 (6.4)/ 59.7 (8.9)	Angiography (≥1 coronary vessel with >50% stenosis); ≤55 for males and ≤60 for females.	Angiography normal, >45 yrs	21239051
**MIGen**	1274/1407	100.0	37.2	39.9	42.4 (6.6)/ 43.0 (7.8)*	MI (men <50 yrs / women <60 yrs)	Hospital-based, community based, or nested case-control	19198609
**OHGS1**	1542/1455	61.6	24.1	48.0	48.7 (7.3)/ 75.9 (5.0)	Angiographic (>50% stenosis)	Asymptomatic	19371834
**PennCATH**	933/468†	50.3	23.7	51.9	52.7 (7.6)/ 61.7 (9.6)	Angiography (≥1 coronary vessel with >50% stenosis); ≤60 for males and ≤65 for females.	Angiography normal, men >40 yrs / women >45 yrs	19198609; 17258089
**WTCCC‡**	1926/2938	71.5	20.7	50.0	49.8 (7.7)/‡	Validated MI, CABG, PTCA or angina with positive non-invasive testing <66 yrs	Unselected	17554300

M(SD) = mean (standard deviation); AMI = acute myocardial infarction; CABG = coronary artery bypass surgery; CAD = coronary artery disease; CVA = cerebrovascular accident; CHD = coronary heart disease; ECG = electrocardiogram; MI = myocardial infarction; NSTEMI = Non-ST-Elevated Myocardial Infarction; PTCA = percutaneous transluminal coronary angioplasty; STEMI = ST segment elevation myocardial infarction; PAD = peripheral artery disease; PCI = percutaneous coronary intervention;

*for cases age at diagnosis; for controls age at recruitment

†Cases: Angiographic CAD (>50% stenosis in at least 1 vessel); controls: Angiography normal or <10% stenosis in all vessels.

‡WTCC controls comprised of an equal number of subjects from the 1958 Birth Cohort and from the National Blood Service (NBS) Donors. The latter were recruited in equal 10 years age bands from 11 to 70 years of age. Additional phenotypes are not available for these controls.

Table e3. List of independent SNPs (r^2 <0.05) with nominal association (p<0.01) to both CAD and migraine.

SNP	Chr	Position*	CAD P value	Migraine P value	Direction of effect†	Nearest gene§
rs13208321	6	96967075	2.53E-03	1.41E-10	+	FUT9, UFL1
rs11759769	6	97171933	1.49E-03	1.71E-10	+	FHL5
rs9349379	6	13011943	8.97E-08	5.88E-09	+	PHACTR1
rs11000137	10	53351800	2.31E-03	7.99E-07	+	PRKG1
rs7905968	10	105125309	9.00E-03	3.65E-06	-	TAF5
rs9490306	6	121858578	1.53E-03	4.80E-06	+	GJA1, HSF2
rs1890185	10	104738708	1.92E-03	5.58E-06	+	CNNM2
rs7920251	10	104940187	2.60E-03	1.25E-05	-	NT5C2
rs10456100	6	39291448	5.16E-04	2.37E-05	+	KCNK5
rs11986252	8	138699854	8.35E-03	2.49E-05	-	LOC101927915, FAM135B
rs11079844	17	44383333	7.30E-06	3.06E-05	+	SNF8, GIP
rs1234221	10	89606459	6.96E-03	5.07E-05	-	CFL1P1, KLLN
rs12181638	6	121855958	1.36E-03	6.29E-05	+	GJA1, HSF2
rs17453089	1	231224476	9.93E-03	1.03E-04	+	PCNXL2
rs9992598	4	20208689	9.65E-03	1.05E-04	-	SLIT2
rs3010671	9	80267958	9.86E-03	1.52E-04	+	PSAT1, LOC101927450
rs10859876	12	94282757	9.59E-03	2.06E-04	+	MIR3685, METAP2
rs2015278	12	67154556	5.20E-03	2.07E-04	-	LOC100507195, RAP1B
rs6469372	8	89443032	1.84E-03	2.35E-04	-	MMP16, RIPK2
rs10794612	10	124501636	1.93E-03	2.36E-04	+	C10orf120, DMBT1P1
rs7136874	12	110598041	2.49E-03	2.49E-04	-	BRAP
rs12951376	17	17693534	2.30E-04	2.56E-04	+	TOM1L2
rs12917651	16	74024197	1.55E-03	2.64E-04	-	CFDP1
rs7908000	10	69689901	7.87E-03	3.03E-04	-	ATOH7, PBLD
rs7304572	12	111009456	1.52E-03	3.09E-04	-	NAA25
rs9856543	3	106074814	6.52E-03	3.55E-04	-	NONE, ALCAM
rs7302763	12	110311952	6.18E-04	3.56E-04	-	FAM109A, SH2B3
rs11066322	12	111406912	2.64E-03	3.91E-04	+	PTPN11
rs17015634	2	78855471	5.12E-03	4.14E-04	+	LOC101927967, REG3G
rs1889277	6	72386917	3.26E-04	4.15E-04	-	LINC00472, RIMS1
rs4302641	6	71124890	6.54E-03	4.23E-04	+	COL9A1, LOC101928353
rs4783386	16	21139552	6.60E-03	4.58E-04	-	ZP2, ANKS4B
rs7749094	6	159767271	4.50E-03	5.52E-04	+	LOC102724053, SOD2
rs17043141	12	76149365	6.63E-04	6.78E-04	+	E2F7, NAV3
rs17801974	5	150617527	3.84E-03	7.16E-04	-	GM2A
rs10096696	8	4027418	8.90E-03	7.38E-04	+	CSMD1
rs13270676	8	74490846	7.59E-03	7.79E-04	-	LOC101926926, STAU2-AS1
rs12423041	12	110809229	4.55E-03	8.54E-04	-	MAPKAPK5
rs10109848	8	58007616	1.59E-03	8.89E-04	-	LINC00968, IMPAD1

Table e3 (continued)

SNP	Chr	Position*	CAD P value	Migraine P value	Direction of effect†	Nearest gene§
rs7920106	10	7393139	4.89E-03	9.05E-04	-	SFMBT2
rs2664283	10	75296379	1.03E-03	1.05E-03	-	CAMK2G
rs7818729	8	127474852	6.48E-03	1.05E-03	-	LOC101927657, FAM84B
rs16906779	9	119959632	7.33E-03	1.07E-03	-	TLR4, BRINP1
rs4925138	17	17933518	5.21E-03	1.07E-03	+	DRG2
rs7947345	11	74031875	3.34E-03	1.09E-03	-	POLD3
rs12540319	7	27463119	8.58E-03	1.11E-03	-	EVX1-AS, HIBADH
rs1815519	1	100857130	5.81E-03	1.19E-03	+	GPR88, LINC01349
rs10828092	10	20962552	2.80E-03	1.21E-03	+	MIR4675, NEBL
rs7311376	12	109089942	1.78E-03	1.22E-03	+	IFT81
rs11191841	10	105629601	6.12E-03	1.27E-03	+	OBFC1
rs3729702	10	74900641	6.24E-03	1.44E-03	-	PPP3CB
rs2511439	11	67610611	5.95E-03	1.48E-03	-	CHKA
rs765467	6	72181890	2.06E-07	1.50E-03	-	LINC00472
rs11195781	10	113743177	2.88E-03	1.55E-03	-	ADRA2A, GPAM
rs17465651	1	38191083	2.80E-04	1.62E-03	+	INPP5B, SF3A3
rs2365261	1	61810709	1.40E-03	1.80E-03	+	NFIA, MGC34796
rs2039623	13	39637964	7.78E-03	1.85E-03	+	COG6, LINC00332
rs10916649	1	222835688	3.44E-04	1.97E-03	-	WDR26, CNIH3
rs7963504	12	109391263	2.28E-03	1.98E-03	-	FAM216A
rs7773293	6	114070787	4.61E-04	1.98E-03	-	LOC101927686
rs2476601	1	114179091	7.33E-04	2.05E-03	-	PTPN22
rs8014986	14	99205471	3.02E-05	2.20E-03	+	HHIPL1
rs11120900	1	7300767	9.35E-03	2.20E-03	-	CAMTA1
rs10520569	15	82520393	3.65E-03	2.21E-03	-	ADAMTSL3, EFTUD1P1
rs838717	2	233961183	2.49E-03	2.24E-03	-	DGKD
rs488624	9	27205792	5.40E-03	2.49E-03	+	TEK
rs10786736	10	104839106	4.08E-05	2.54E-03	-	NT5C2
rs13200037	6	13038738	7.96E-04	2.56E-03	+	PHACTR1
rs1427461	4	83460158	1.15E-03	2.71E-03	-	RASGEF1B, HNRNPD
rs2929282	15	42033223	3.07E-03	2.84E-03	-	FRMD5
rs1516133	2	188590478	5.48E-03	2.91E-03	-	TFPI, GULP1
rs3904265	11	50316695	6.04E-03	2.94E-03	-	LOC441601, LOC646813
rs12941722	17	19292774	3.97E-03	2.97E-03	-	RNF112, SLC47A1
rs12450679	17	8297718	3.30E-04	3.01E-03	+	NDEL1
rs2929275	15	42046189	4.25E-03	3.05E-03	+	FRMD5
rs10518874	15	34446099	7.81E-03	3.06E-03	-	MIR4510, C15orf41
rs7501148	16	71875058	3.88E-04	3.24E-03	+	C16orf47, LOC100506172
rs17476034	11	50056843	8.67E-04	3.31E-03	+	OR4C12, LOC441601
rs9579648	13	30225032	2.09E-03	3.37E-03	+	ALOX5AP
rs10945970	6	164617709	2.33E-03	3.51E-03	-	QKI, C6orf118

Table e3 (continued)

SNP	Chr	Position*	CAD P value	Migraine P value	Direction of effect†	Nearest gene§
rs11639989	16	9682661	4.39E-03	3.55E-03	+	MIR7641-2, GRIN2A
rs13263450	8	89643491	1.17E-03	3.59E-03	+	MMP16, LOC101929709
rs1316410	1	228952429	2.04E-03	3.71E-03	+	CAPN9
rs7108904	11	51334382	3.71E-03	3.81E-03	-	OR4A5, OR4C46
rs12636192	3	151015598	3.50E-03	3.96E-03	-	RNF13
rs4026627	1	210924971	6.32E-03	4.05E-03	+	FAM71A, BATF3
rs8188911	11	50727590	2.09E-03	4.25E-03	+	LOC646813, OR4A5
rs2280134	11	3638095	3.05E-04	4.32E-03	-	ART1
rs10763976	10	34604298	1.14E-03	4.43E-03	-	PARD3
rs827782	5	169766421	6.40E-03	4.51E-03	+	CTD-2270F17.1
rs7029433	9	83423900	3.25E-03	4.54E-03	+	TLE1
rs10902284	11	51078592	2.09E-03	4.63E-03	+	LOC646813, OR4A5
rs13231	6	161059847	2.35E-04	4.87E-03	+	PLG
rs1508063	2	72957384	2.09E-03	4.91E-03	+	EXOC6B, SPR
rs1108927	1	34105854	4.78E-03	4.93E-03	+	CSMD2
rs1826907	4	100520149	6.07E-04	4.94E-03	+	ADH1C, ADH7
rs7281595	21	42389601	8.35E-03	4.96E-03	+	UMODL1
rs6069057	20	36240223	2.58E-03	5.10E-03	+	TGM2, KIAA1755
rs11187393	10	95097418	1.14E-03	5.12E-03	-	MYOF
rs2925174	5	114680083	4.24E-03	5.29E-03	-	CCDC112, FEM1C
rs1556581	1	43544777	6.15E-03	5.33E-03	-	TIE1
rs346522	19	48954385	4.99E-04	5.43E-03	+	SMG9, KCNN4
rs2068414	11	49658664	5.19E-03	5.59E-03	+	LOC440040
rs4692549	4	26194979	3.23E-03	5.61E-03	+	TBC1D19
rs924765	8	134917078	4.34E-03	5.78E-03	-	LOC101927798, LOC101927822
rs12083075	1	167377625	6.38E-03	5.83E-03	+	NME7
rs1230666	1	113974933	5.54E-04	5.91E-03	-	MAGI3
rs2300435	10	124242269	7.44E-03	5.92E-03	+	HTRA1
rs196569	7	36270857	6.60E-03	5.99E-03	-	EEPD1
rs1442243	7	52615087	1.74E-03	6.09E-03	-	COBL, POM121L12
rs17605951	16	70855143	8.10E-03	6.16E-03	-	PMFBP1, ZFHX3
rs4479352	18	60066408	9.59E-03	6.23E-03	+	LINC01538, LOC284294
rs11569557	19	6631510	9.11E-03	6.29E-03	+	C3
rs371100	4	171708106	5.66E-03	6.51E-03	-	LOC101928223, LOC100506122
rs693720	11	77570200	5.70E-03	6.74E-03	+	KCTD21
rs12130112	1	37979629	7.46E-03	6.76E-03	-	EPHA10
rs2478516	1	228935275	5.62E-03	7.03E-03	+	AGT, CAPN9
rs5768001	22	46576776	4.77E-04	7.06E-03	-	LOC284930
rs11929794	4	180109117	8.22E-03	7.62E-03	-	LINC01098, NONE

Table e3 (continued)

SNP	Chr	Position*	CAD P value	Migraine P value	Direction of effect†	Nearest gene§
rs17278009	2	55539888	5.32E-04	7.64E-03	+	CCDC88A, CFAP36
rs12989712	2	201814536	4.48E-03	7.67E-03	-	CASP8
rs6694531	1	148950136	1.52E-04	7.79E-03	-	HORMAD1
rs6920844	6	42653616	4.24E-03	7.83E-03	-	UBR2
rs7662464	4	155905830	2.03E-03	7.94E-03	-	LRAT, RBM46
rs17193778	3	1797161	4.38E-04	7.98E-03	-	CNTN6, CNTN4
rs2042549	2	169238400	4.26E-04	8.09E-03	-	CERS6
rs12073872	1	170817021	3.49E-03	8.17E-03	+	SUCO
rs6832419	4	40096367	2.90E-03	8.25E-03	-	CHRNA9, RBM47
rs7310409	12	119909244	4.15E-06	8.44E-03	+	HNF1A
rs7990504	13	41498003	3.03E-04	8.46E-03	-	VWA8-AS1, DGKH
rs12275064	11	49109173	8.06E-05	8.47E-03	+	TRIM64C, FOLH1
rs10137574	14	99232677	1.35E-03	8.70E-03	-	CYP46A1
rs6139515	20	4595512	5.14E-03	8.70E-03	-	ADRA1D, PRNP
rs11941167	4	43817331	1.84E-03	8.92E-03	-	GRXCR1, KCTD8
rs10490445	2	37470988	7.95E-03	8.96E-03	+	QPCT, CDC42EP3
rs17642516	6	148858197	4.93E-03	9.05E-03	+	SASH1
rs293974	11	26546702	6.79E-03	9.11E-03	+	ANO3, MUC15
rs550517	5	31421693	8.42E-03	9.14E-03	-	CDH6, DROSHA
rs7096797	10	43640310	1.50E-03	9.27E-03	+	HNRNPA3P1, LINC00619
rs11694529	2	49114653	8.52E-03	9.34E-03	+	FSHR
rs934287	2	203416552	3.93E-08	9.49E-03	+	ICA1L
rs2160847	2	211309361	5.96E-03	9.54E-03	-	CPS1, ERBB4
rs10050682	5	133358825	9.98E-03	9.71E-03	+	VDAC1
rs7095475	10	106197923	4.84E-03	9.75E-03	-	CFAP58
rs12898704	15	72048036	1.79E-03	9.87E-03	+	LOXL1, STOML1
rs10142643	14	29490756	8.58E-03	9.92E-03	-	PRKD1, G2E3

CAD = coronary artery disease. *Positions refer to build NCBI36/hg18. †SNPs with the same effect direction for association to CAD and migraine are marked as plus (+), opposing effect direction are marked as minus (-). §RefSeq genes. For intergenic SNPs the nearest gene on either side is listed.
The list is sorted by increasing *P* value for association to migraine.

Table e4. Association between coronary artery disease polygenic risk score and the presence of migraine.

	CAD risk SNP set*	OR	Standard error	R^2 explained†	P value
All migraine	Weak	1.000	0.0005	1.93E-06	8.56E-01
	Moderate	0.997	0.0012	4.27E-04	6.89E-03
	Strong	0.997	0.0017	1.76E-04	8.28E-02
Migraine without aura	Weak	1.000	0.0007	5.88E-05	5.11E-01
	Moderate	0.994	0.0017	1.96E-03	1.48E-04
	Strong	0.992	0.0023	1.64E-03	5.13E-04
Migraine with aura	Weak	1.000	0.0007	1.95E-05	6.74E-01
	Moderate	1.000	0.0018	5.17E-08	9.83E-01
	Strong	1.003	0.0024	1.37E-04	2.64E-01

CAD = coronary artery disease.

*Weak – *P* value <1.0E-02; Moderate – *P* value <1.0E-04; Strong – *P* value <5.0E-08.

†Difference in Nagelkerke's pseudo R^2 between model including and model not including CAD polygenic risk score.

The CAD risk SNP sets were based on SNPs with weak ($P<1 \times 10^{-2}$), moderate ($P<1 \times 10^{-4}$) or strong ($P<5 \times 10^{-8}$) association to CAD in the CAD study.

Analyses were corrected for gender, and for dummy-coded covariates representing the individual migraine cohorts.

Table e5. Details of lead SNPs for coronary artery disease and migraine at each overlapping locus identified by Cross-Phenotype Spatial Mapping (CPSM).

**Locus no.**	**Chr band**		**SNP**	**Position***	**CAD *P* value†**	**Migraine *P* value†**	**Migraine without aura *P* value†**	**Migraine with aura *P* value†**	**Direction of effect‡**	**Nearest gene§**
1	6p24	Lead CAD SNP	rs4714955	13,011,421	**9.80E-11**	5.04E-07	**2.68E-09**	3.43E-01	+---	*PHACTR1*
		Lead migraine SNP	rs9349379	13,011,943	8.97E-08	**5.88E-09**	**2.52E-10**	2.36E-01	+---	*PHACTR1*
2	17q21	Lead CAD SNP	rs46522	44,343,596	2.58E-07	1.24E-04	6.93E-03	8.10E-02	+---	*UBE2Z*
		Lead migraine SNP	rs11079844	44,383,333	7.30E-06	3.06E-05	8.30E-03	1.57E-02	+---	*SNF8*, *GIP*
3	6q16	Lead CAD SNP	rs2064947	97,159,475	6.40E-04	2.46E-07	**5.47E-11**	2.78E-02	+---	*FHL5*
		Lead migraine SNP	rs13208321	96,967,075	2.53E-03	**1.41E-10**	**1.35E-12**	7.40E-04	+---	*FUT9*, *UFL1*
4	12q24	Lead CAD SNP	rs2238151	110,696,216	2.43E-07	7.29E-03	2.34E-03	3.95E-01	+---	*ALDH2*
		Lead migraine SNP	rs7136874	110,598,041	2.49E-03	2.49E-04	1.26E-03	1.05E-01	+---	*BRAP*
5	17p11	Lead CAD SNP	rs6502622	17,775,416	7.61E-06	3.03E-03	4.88E-03	5.31E-01	++++	*TOM1L2*
		Lead migraine SNP	rs12951376	17,693,534	2.30E-04	2.56E-04	1.23E-01	8.55E-02	++++	*TOM1L2*
6	16q23	Lead CAD SNP	rs4888422	74,028,077	6.88E-05	9.55E-04	7.38E-02	2.59E-02	+---	*CFDP1*, *TMEM170A*
		Lead migraine SNP	rs12917651	74,024,197	1.55E-03	2.64E-04	2.07E-02	6.13E-02	+---	*CFDP1*
7	10q24	Lead CAD SNP	rs10786736	104,839,106	4.08E-05	2.54E-03	5.28E-02	8.97E-01	+--+	*NT5C2*
		Lead migraine SNP	rs7905968	105,125,309	9.00E-03	3.65E-06	1.68E-02	8.62E-03	+---	*TAF5*
8	2q33	Lead CAD SNP	rs3845800	203,442,610	**4.47E-11**	2.90E-02	3.32E-02	9.53E-01	+---	*ICA1L*

		Lead migraine SNP	rs934287	203,416,552	**3.93E-08**	9.49E-03	8.91E-03	7.92E-01	+---	*ICA1L*
9	10q24	Lead CAD SNP	rs11191425	104,615,960	1.16E-05	2.83E-02	4.88E-02	7.55E-01	+---	*C10orf32-AS3MT*
		Lead migraine SNP	rs1890185	104,738,708	1.92E-03	5.58E-06	2.21E-02	9.18E-03	+---	*CNNM2*
10	6q13	Lead CAD SNP	rs9351814	72,250,428	**9.90E-09**	7.51E-03	3.47E-03	7.52E-02	++++	*LINC00472*, *RIMS1*
		Lead migraine SNP	rs1889277	72,386,917	3.26E-04	4.15E-04	2.47E-02	6.64E-02	++++	*LINC00472*, *RIMS1*
11	8q21	Lead CAD SNP	rs1580508	89,639,576	9.40E-04	3.95E-03	1.25E-07	3.60E-01	++++	*MMP16*, *RIPK2*
		Lead migraine SNP	rs716881	89,585,557	3.29E-03	1.44E-03	2.36E-07	3.81E-01	++++	*MMP16*, *RIPK2*
12	19q13	Lead CAD SNP	rs2231940	46,636,077	4.13E-03	2.07E-04	5.51E-04	6.14E-01	+---	*ATP5SL*
		Lead migraine SNP	rs4803455	46,543,349	8.56E-01	1.80E-07	5.47E-05	6.42E-03	+---	*TGFB1*
13	12q24	Lead CAD SNP	rs871921	109,599,496	6.66E-05	1.27E-02	8.82E-04	3.43E-01	++++	*HVCN1*
		Lead migraine SNP	rs3864937	109,567,467	6.98E-01	8.12E-04	8.48E-02	3.71E-03	+---	*TCTN1*
14	8q21	Lead CAD SNP	rs7004601	89,440,710	2.32E-04	1.66E-03	1.78E-06	3.83E-01	++++	*MMP16*, *RIPK2*
		Lead migraine SNP	rs6469372	89,443,032	1.84E-03	2.35E-04	3.66E-07	3.70E-01	++++	*MMP16*, *RIPK2*
15	16q24	Lead CAD SNP	rs12924776	88,114,093	1.80E-06	5.73E-02	8.79E-01	4.13E-02	++-+	*SPG7*
		Lead migraine SNP	rs7359417	88,144,059	6.60E-2	6.65E-04	2.53E-02	3.60E-02	++++	*SPG7*
16	9p21	Lead CAD SNP	rs190661	23,444,661	2.57E-03	3.05E-03	1.27E-02	4.59E-01	+---	*FLJ35282*, *ELAVL2*
		Lead migraine SNP	rs274934	23,476,403	1.00E-02	2.37E-06	9.01E-05	2.40E-01	+---	*FLJ35282*, *ELAVL2*

CAD = coronary artery disease. *Positions refer to build NCBI36/hg18. †*P* values <5.0 x10^{-8} are highlighted in bold. ‡Direction of effect for CAD, migraine, migraine without aura and migraine with aura respectively. Direction of effect for association to CAD is set as positive (+). §RefSeq genes. For intergenic SNPs the nearest gene on either side is listed.

Table e6. Functional variants (non-synonymous or splice site variants) in LD (r^2>0.8) with lead SNPs at overlapping loci.

Locus no.	Chr band		Lead SNP	Nearest gene to lead SNP‡	Proxy SNP	Distance from lead SNP	Gene*	r^2	D'	Coding change	Type
2	17q21	Lead CAD SNP	rs46522	UBE2Z	rs2291725	50535	*GIP*	0.94	1.0	p.Ser103Gly	non-synonymous
					rs2291726	50657	*GIP*	0.91	1.0	c.258-73A>G	splice-site
		Lead migraine SNP	rs11079844	SNF8, GIP	rs2291725	10798	*GIP*	0.93	0.97	p.Ser103Gly	non-synonymous
					rs2291726	10920	*GIP*	0.97	1.0	c.258-73A>G	splice-site
8	2q33	Lead CAD SNP	rs3845800	ICA1L	rs35212307	31391	*WDR12*	1.0	1.0	p.Ile75Val	non-synonymous
					rs72932557	112452	*ALS2CR8*	1.0	1.0	p.Tyr571Phe	non-synonymous
9	10q24	Lead CAD SNP	rs11191425	C10orf32-AS3MT	rs17115100	-34577	*CYP17A1*	1.0	1.0	c.1140-25C>A	splice-site
12	19q13	Lead CAD SNP	rs2231940	ATP5SL	rs10853751	-41017	*EXOSC5*	1.0	1.0	p.Thr5Met	non-synonymous
					rs284662	-11962	*B3GNT8*	0.91	1.0	p.Ser137Gly	non-synonymous
					rs1043413	-4940	*ATP5SL*	1.0	1.0	p.[Cys165Ser, Cys132Ser, Cys159Ser]	non-synonymous
					rs2231940	0	*ATP5SL*	1.0	1.0	p.[Asn40Ser, Asn34Ser]	non-synonymous
15	16q24	Lead migraine SNP	rs7359417	SPG7	rs2292954	-3435	*SPG7*	1.0	1.0	p.Thr503Ala	non-synonymous
					rs12960	3770	*SPG7*	0.94	1.0	p.Arg688Gln	non-synonymous

CAD = coronary artery disease.

*RefSeq genes. For intergenic SNPs the nearest gene on either side is listed.

LD calculations are based on 1000 genomes phase 1 CEU

Table e7. Expression quantitative trait loci (eQTLs) at overlapping loci determined from gene expression in peripheral blood.

Locus no.	Chr band	Locus SNP	eQTL SNP	eQTL *P* value*	eQTL probe	eQTL gene	*P* value for correlation with eQTL locus†
2	17q21	rs11079844	rs1985785	1.94E-26	11745885_a_at	UBE2Z	3.18E-03
2	17q21	rs11079844	rs4597361	5.39E-08	11715988_x_at	ATP5G1	0.90
4	12q24	rs7136874	rs668774	4.74E-11	11731248_a_at	TMEM116	1.0
4	12q24	rs7136874	rs7956495	1.95E-06	11743486_a_at	NAA25	1.0
5	17p11	rs12951376	rs6502629	5.26E-06	11758738_a_at	SREBF1	0.18
15	16q24	rs7359417	rs2377056	2.10E-46	11725404_x_at	RPL13	1.0

The table shows eQTLs at which the credible set of causative SNPs at the eQTL locus intersect with the credible set of causative SNPs at the overlap locus, represented by the migraine dataset.

**P* value for association between eQTL SNP and expression of the eQTL probe.

†*P* value for a test of significant correlation between association z-scores in the migraine credible set compared to the overlapping eQTL credible set. Correlation was measured using Pearson's correlation coefficient and significance assessed by a two-tailed t-test with n-2 degrees of freedom, where n is the number of SNPs overlapping in the credible sets. The final p-values given here are Bonferroni corrected for 6 tests.
