# Supplementary material for: Genetic analysis for a shared biological basis between migraine and coronary artery disease
Source: Neurol Genet. 2015 Jul 2;1(1):e10. doi: 10.1212/NXG.0000000000000010 (PMC4821079; doi:10.1212/NXG.0000000000000010)
Supplement: Coinvestigators [file supp_1_1_e10_v2_index.html]

Coinvestigators 

# Genetic analysis for a shared biological basis between migraine and coronary artery disease

## Coinvestigators

**Files in this Data Supplement:**

- Coinvestigators - PDF
