# Supplementary material for: Genetic analysis for a shared biological basis between migraine and coronary artery disease
Source: Neurol Genet. 2015 Jul 2;1(1):e10. doi: 10.1212/NXG.0000000000000010 (PMC4821079; doi:10.1212/NXG.0000000000000010)
Supplement: Coinvestigators [file supp_1.1.e10_Coinvestigators.pdf]

Consortium membership

The International Headache Genetics Consortium

Consortium members listed by their main affiliated cohort:

AGES: Leonore Launer[1]

ALSPAC: George Davey Smith[2], George McMahon[2]

Australia ATM: Dale Nyholt[3]

Barcelona headache group: Alfons Macaya[4], Patricia Pozo-Rosich[5], Bru Cormand[6], Jessica Fernandez[5], Marta Vila-Pueyo[4], Celia Sintas[6]

Danish Headache Center, Glostrup Hospital: Jes Olesen[2], Anne Francke Christensen[2], Ann-Louise Esserlind[2]

ERF: Najaf Amin[7]

Estonian Biobank: Tonu Esko[8]

Finnish MA: Aarno Palotie[9], Mikko Kallela[10], Maija Wessman[11], Ville Artto[10], Verneri Anttila[12], Eija Hämäläinen[13], Priit Palta[13], Padhraig Gormley[9], Ester Cuenca[9]

FinnTwin: Jaakko Kaprio[13]

German MO/MA: Martin Dichgans[14], Hartmut Göbel[15], Christian Kubisch[16], Tobias Freilinger[17], Rainer Malik[14], Bertram Muller-Myhsok[18]

HUNT: John-Anker Zwart[19], Bendik Winsvold[19], Line Jacobsen[19], Linda Pedersen[19]

Kaiser Permanente: Alice Pressman[20]

LUMINA MO/MA: Arn van den Maagdenberg[21], Gisela Terwindt[22], Boukje de Vries[21], Rune R. Frants[21], Michel Ferrari[22]

NTR/NESDA: Dorret I. Boomsma[23], Lannie Ligthart[23], Brenda Penninx[24]

NFBC1966: Marjo-Riitta Jarvelin[25], Markku Koiranen[26]

Rotterdam III: Cornelia van Duijn[7] , M Arfan Ikram[7]

Swedish Twin Registry: Andrea Carmine Belin[27], Nancy Pedersen[28]

TWINS UK: Lynn Cherkas[29], Lydia Quaye[29]

WGHS: Daniel Chasman[30], Tobias Kurth[31], Markus Schuerks[32]

Young Finns: Terho Lehtimaki[33], Olli Raitakari[34]

23&Me, Mountainview, California: Nick Eriksson[35]

1. Laboratory of Epidemiology, Demography and Biometry, National Institute on Aging, Bethesda, Maryland, USA.
2. Medical Research Council (MRC) Integrative Epidemiology Unit at the University of Bristol, Bristol, UK.
3. Queensland Institute of Medical Research, Brisbane, Queensland, Australia.
4. Pediatric Neurology Research Group, Institut de Recerca (VHIR), Universitat Autònoma de Barcelona, Barcelona, Spain.
5. Headache and Neurological Pain Research Group, Institut de Recerca (VHIR), Universitat Autònoma de Barcelona, Barcelona.
6. Departament de Genètica, Facultat de Biologia, Universitat de Barcelona, Barcelona, Spain.
7. Department of Epidemiology, Erasmus University Medical Centre, Rotterdam, The Netherlands.
8. Estonian Genome Center, University of Tartu, Tartu, Estonia.
9. Program in Medical and Population Genetics, Broad Institute of Harvard and MIT, Cambridge, MA, USA.
10. Department of Neurology, Helsinki University Central Hospital, Helsinki, Finland.
11. Institute of Genetics, Folkhälsan Research Center, Helsinki, Finland.
12. Analytic and Translational Genetics Unit, Massachusetts General Hospital, Boston, MA, USA.
13. Institute for Molecular Medicine Finland (FIMM), University of Helsinki, Helsinki, Finland.
14. Institute for Stroke and Dementia Research, Klinikum der Universität München, Ludwig-Maximilians-Universität, Munich, Germany.
15. Kiel Pain and Headache Center, Kiel, Germany.
16. Institute of Human Genetics, University of Ulm, Ulm, Germany.
17. Department of Neurology and Epileptology and Hertie-Institute for Clinical Brain Research, University of Tübingen.
18. Max Planck Institute of Psychiatry, Munich, Germany.
19. FORMI, Oslo University Hospital, Oslo, Norway.
20. Division of Research, Kaiser Permanente, Oakland, CA, USA.
21. Department of Human Genetics, Leiden University Medical Centre, Leiden, The Netherlands.
22. Department of Neurology, Leiden University Medical Centre, Leiden, The Netherlands.
23. Department of Biological Psychology, VU University, Amsterdam, The Netherlands.
24. Department of Psychiatry, VU University Medical Center, Amsterdam, The Netherlands.
25. Biocenter Oulu, University of Oulu, Oulu, Finland.
26. Institute of Health Sciences, University of Oulu, Oulu, Finland.
27. Department of Neuroscience, Karolinska Institutet, Stockholm, Sweden.
28. Department of Medical Epidemiology and Biostatistics, Karolinska Institutet, Stockholm, Sweden.
29. Department of Twin Research and Genetic Epidemiology, King's College London, London, UK.
30. Division of Preventive Medicine, Brigham and Women's Hospital, Harvard Medical School, Boston, MA, USA.
31. Institut National de la Santé et de la Recherche Médicale (INSERM) Research Center for Epidemiology and Biostatistics (U897) Team–Neuroepidemiology, Bordeaux, France.
32. Department of Neurology, University Hospital Essen, Essen, Germany.
33. Department of Clinical Chemistry, Fimlab Laboratories, Tampere, Finland.
34. Department of Clinical Physiology and Nuclear Medicine, Turku University Hospital, Turku, Finland.
35. 23andMe, Mountain View, California, USA.

The CARDIoGRAM Consortium

ADVANCE: Devin Absher[1], Themistocles L. Assimes[2], Stephen Fortmann[2], Alan Go[3], Mark Hlatky[2], Carlos Iribarren[3], Joshua Knowles[2], Richard Myers[1], Thomas Quertermous[2], Steven Sidney[3], Neil Risch[4], Hua Tang[5]

CADomics: Stefan Blankenberg[6], Tanja Zeller[6], Arne Schillert[7], Philipp Wild[6], Andreas Ziegler[7], Renate Schnabel[6], Christoph Sinning[6], Karl Lackner[8], Laurence Tiret[9], Viviane Nicaud[9], Francois Cambien[9], Christoph Bickel[6], Hans J. Rupprecht[6], Claire Perret[9], Carole Proust[9], Thomas Münzel[6]

CHARGE: Maja Barbalic[10], Joshua Bis[11], Eric Boerwinkle[12], Ida Yii-Der Chen[13], L. Adrienne Cupples[14], Abbas Dehghan[15], Serkalem Demissie-Banjaw[16], Aaron Folsom[17], Nicole Glazer[18], Vilmundur Gudnason[19], Tamara

Harris[20], Susan Heckbert[21], Daniel Levy[22], Thomas Lumley[23], Kristin Marciante[24], Alanna Morrison[25], Christopher J. O'Donnell[26], Bruce M. Psaty[27], Kenneth Rice[23], Jerome I. Rotter[13], David S. Siscovick[28], Nicholas Smith[21], Albert Smith[29], Kent D. Taylor[13], Cornelia van Duijn[15], Kelly Volcik[25], Jaqueline Whitteman[15], Vasan Ramachandran[30], Albert Hofman[30], Andre Uitterlinden[30]

deCODE: Solveig Gretarsdottir[31], Jeffrey R. Gulcher[31], Hilma Holm[31], Augustine Kong[31], Kari Stefansson[31], Gudmundur Thorgeirsson[32], Karl Andersen[32], Gudmar Thorleifsson[31], Unnur Thorsteinsdottir[31]

GERMIFS I and II: Jeanette Erdmann[33], Marcus Fischer[34], Anika Grosshennig[7], Christian Hengstenberg[34], Inke R. König[7], Wolfgang Lieb[35], Patrick Linsel-Nitschke[36], Michael Preuss[7], Klaus Stark[34], Stefan Schreiber[37], H.-Erich Wichmann[38], Andreas Ziegler[7], Heribert Schunkert[39]

GERMIFS III (KORA): Zouhair Aherrahrou[36], Petra Bruse[36], Angela Doering[38], Jeanette Erdmann[33], Christian Hengstenberg[34], Thomas Illig[38], Norman Klopp[38], Inke R. König[7], Patrick Linsel-Nitschke[36], Christina Loley[7], Anja Medack[36], Christina Meisinger[38], Thomas Meitinger[40], Janja Nahrstaedt[7], Annette Peters[38], Michael Preuss[7], Klaus Stark[34], Arnika K. Wagner[36], H.-Erich Wichmann[38], Christina Willenborg[33], Andreas Ziegler[7], Heribert Schunkert[39]

LURIC/AtheroRemo: Bernhard O. Böhm[41], Harald Dobnig[42], Tanja B. Grammer[43], Eran Halperin[44], Michael M. Hoffmann[45], Marcus Kleber[46], Reijo Laaksonen[47], Winfried März[43], Andreas Meinitzer[48], Bernhard R. Winkelmann[49], Stefan Pilz[42], Wilfried Renner[48], Hubert Scharnagl[48], Tatjana Stojakovic[48], Andreas Tomaschitz[42], Karl Winkler[45]

MIGen: Benjamin F. Voight[50], Kiran Musunuru[51], Candace Guiducci[51], Noel Burtt[51], Stacey B. Gabriel[51], David S. Siscovick[28], Christopher J. O'Donnell[26], Roberto Elosua[52], Leena Peltonen[53], Veikko Salomaa[54], Stephen M. Schwartz[28], Olle Melander[55], David Altshuler[56], Sekar Kathiresan[57]

OHGS: Alexandre F. R. Stewart[58], Li Chen[58], Sonny Dandona[58], George A. Wells[58], Olga Jarinova[58], Ruth McPherson[58], Robert Roberts[58]

PennCATH/MedStar: Muredach P. Reilly[59], Mingyao Li[60], Liming Qu[60], Robert Wilensky[61], William Matthai[61], Hakon H. Hakonarson[62], Joe Devaney[63], Mary Susan Burnett[63], Augusto D. Pichard[63], Kenneth M. Kent[63], Lowell Satler[63], Joseph M. Lindsay[63], Ron Waksman[63], Christopher W. Knouff[64], Dawn M. Waterworth[64], Max C. Walker[64], Vincent Mooser[64], Stephen E. Epstein[63], Daniel J. Rader[59]

WTCCC: Nilesh J. Samani[65], John R. Thompson[66], Peter S. Braund[65], Christopher P. Nelson[65], Benjamin J. Wright[66], Anthony J. Balmforth[67], Stephen G. Ball[67], Alistair S. Hall[68]

1. Hudson Alpha Institute, Huntsville, Alabama, USA.
2. Department of Medicine, Stanford University School of Medicine, Stanford, California, USA.
3. Division of Research, Kaiser Permanente, Oakland, CA, USA.
4. Institute for Human Genetics, University of California, San Francisco, San Francisco, CA, USA.

5. Department of Genetics, Stanford University School of Medicine, Stanford, CA, USA.
6. Medizinische Klinik und Poliklinik, Universitätsmedizin Mainz, Johannes-Gutenberg Universität Mainz, Mainz, Germany.
7. Institut für Medizinische Biometrie und Statistik, Universität zu Lübeck, Lübeck, Germany.
8. Institut für Klinische Molekularbiologie, Christian-Albrechts Universität, Kiel, Germany.
9. INSERM UMRS 937, Pierre and Marie Curie University (UPMC, Paris 6) and Medical School, Paris, France.
10. University of Texas, Houston, TX, USA.
11. University of Washington, Department of Medicine, Seattle, WA, USA.
12. University of Texas Health Science Center, Human Genetics Center and Institute of Molecular Medicine, Houston, TX, USA.
13. Cedars-Sinai Medical Center, Medical Genetics Institute, Los Angeles, CA, USA.
14. Department of Biostatistics and Epidemiology, Boston University, USA.
15. Department of Epidemiology, Erasmus University Medical Centre, Rotterdam, The Netherlands.
16. Boston University, School of Public Health, Boston, MA, USA.
17. University of Minnesota, Division of Epidemiology and Community Health, Minneapolis, MN, USA.
18. University of Washington, Cardiovascular Health Research Unit and Department of Medicine, Seattle, WA, USA.
19. Icelandic Heart Association/University of Iceland, Research Institute/Faculty of Medicine, Kopavogur/Reykjavik, Iceland.
20. National Institute on Aging, Intramural Research Program, Laboratory of Epidemiology, Demography, and Biometry, Bethesda, MD, USA.
21. University of Washington, Department of Epidemiology, Seattle, WA, USA.
22. National Heart Lung and Blood Institute, Framingham Heart Study, Framingham, MA, USA.
23. University of Washington, Department of Biostatistics, Seattle, WA, USA.
24. University of Washington, Department of Internal Medicine, Seattle, WA, USA.
25. University of Texas, School of Public Health, Houston, TX, USA.
26. National Heart, Lung, and Blood Institute's Framingham Heart Study, Framingham, Massachusetts, USA.
27. Center for Health Studies, Group Health, Departments of Medicine, Epidemiology, and Health Services, Seattle, WA, USA.
28. Cardiovascular Health Research Unit, Departments of Epidemiology and General Medicine, University of Washington, Seattle, WA, USA.
29. Icelandic Heart Association Research Institute, Kopavogur, Iceland.
30. Boston University Medical Center, Boston, MA, USA.
31. deCODE genetics, Reykjavik, Iceland.
32. Department of Medicine, Landspitali University Hospital, 101 Reykjavik, Iceland.
33. Institut für Integrative und Experimentelle Genomik, Universität zu Lübeck, Lübeck, Germany.
34. Klinik und Poliklinik für Innere Medizin II, Universität Regensburg, Regensburg, Germany.
35. Boston University School of Medicine, Framingham Heart Study, Framingham, MA, USA.
36. Medizinische Klinik II, Universität zu Lübeck, Lübeck, Germany.
37. Institut für Klinische Chemie und Laboratoriumsmediizin, Johannes-Gutenberg Universität Mainz, Universitätsmedizin, Mainz, Germany.
38. Institute of Epidemiology, Helmholtz Zentrum München – German Research Center for Environmental Health, Neuherberg, Germany.
39. Deutsches Herzzentrum München, Technische Universität München, Munich, Germany.
40. Institut für Humangenetik, Helmholtz Zentrum München, Deutsches Forschungszentrum für Umwelt und Gesundheit, Neuherberg, Germany.
41. Division of Endocrinology and Diabetes, Graduate School of Molecular Endocrinology and Diabetes, University of Ulm, Ulm, Germany.
42. Division of Endocrinology, Department of Medicine, , Medical University of Graz, Austria.
43. Synlab Center of Laboratory Diagnostics Heidelberg, Heidelberg, Germany.
44. The Blavatnik School of Computer Science and the Department of Molecular Microbiology and Biotechnology, Tel-Aviv University, Tel-Aviv, Israel, and the International Computer Science Institute, Berkeley, CA, USA.
45. Division of Clinical Chemistry, Department of Medicine, Albert Ludwigs University, Freiburg, Germany.
46. LURIC non profit LLC, Freiburg, Germany.
47. Science Center, Tampere University Hospital, Tampere, Finland.
48. Clinical Institute of Medical and Chemical Laboratory Diagnostics, Medical University of Graz, Graz, Austria.
49. Cardiology Group Frankfurt-Sachsenhausen, Frankfurt, Germany.
50. Center for Human Genetic Research, Massachusetts General Hospital, Boston, MA, USA.
51. Cardiovascular Research Center and Cardiology Division, Massachusetts General Hospital, Boston, MA, USA.
52. Cardiovascular Epidemiology and Genetics Group, Institut Municipal d'Investigació Mèdica, Barcelona; Ciber Epidemiología y Salud Pública (CIBERSP), Spain.

53. The Wellcome Trust Sanger Institute, The Wellcome Trust Genome Campus, Hinxton, Cambridge, UK.
54. Chronic Disease Epidemiology Unit, Department of Health Promotion and Chronic Disease Prevention, National Public Health Institute, Helsinki, Finland.
55. Department of Clinical Sciences, Hypertension and Cardiovascular Diseases, University Hospital Malmö, Lund University, Malmö, Sweden.
56. Department of Molecular Biology and Center for Human Genetic Research, Massachusetts General Hospital, Harvard Medical School, Boston, USA.
57. Department of Medicine, Harvard Medical School, Boston, MA, USA.
58. The John and Jennifer Ruddy Canadian Cardiovascular Genetics Centre, University of Ottawa Heart Institute, Ottawa, Ontario, Canada.
59. Institute for Translational Medicine and Therapeutics, Cardiovascular Institute, and Department of Medicine, University of Pennsylvania, Philadelphia.
60. Biostatistics and Epidemiology, University of Pennsylvania, Philadelphia, PA, USA.
61. The Cardiovascular Institute, University of Pennsylvania, Philadelphia, PA, USA.
62. The Center for Applied Genomics, Children's Hospital of Philadelphia, Philadelphia, Pennsylvania, USA.
63. Cardiovascular Research Institute, MedStar Research Institute, Washington Hospital Center, Washington, DC, USA.
64. Genetics Division and Drug Discovery, GlaxoSmithKline, King of Prussia, Pennsylvania 19406, USA.
65. Department of Cardiovascular Sciences, University of Leicester, Clinical Sciences Wing, Glenfield Hospital, Leicester, UK.
66. Department of Health Sciences, University of Leicester, Leicester, UK.
67. LIGHT Research Institute, Faculty of Medicine and Health, University of Leeds, Leeds, UK.
68. Division of Cardiovascular and Neuronal Remodelling, Multidisciplinary Cardiovascular Research Centre, Leeds Institute of Genetics, Health and Therapeutics, University of Leeds, Leeds, UK.
